# Dense Granule Protein GRA64 Interacts with Host Cell ESCRT Proteins during *Toxoplasma gondii* Infection

**DOI:** 10.1128/mbio.01442-22

**Published:** 2022-06-22

**Authors:** Joshua Mayoral, Rebekah B. Guevara, Yolanda Rivera-Cuevas, Vincent Tu, Tadakimi Tomita, Julia D. Romano, Leslie Gunther-Cummins, Simone Sidoli, Isabelle Coppens, Vernon B. Carruthers, Louis M. Weiss

**Affiliations:** a Department of Pathology, Albert Einstein College of Medicine, Bronx, New York, USA; b Department of Anatomy and Structural Biology, Albert Einstein College of Medicine, Bronx, New York, USA; c Departments of Biochemistry, Albert Einstein College of Medicine, Bronx, New York, USA; d Department of Medicine, Albert Einstein College of Medicine, Bronx, New York, USA; e Department of Microbiology and Immunology, University of Michigan Medical School, Ann Arbor, Michigan, USA; f Department of Molecular Microbiology and Immunology, Johns Hopkins Bloomberg School of Public Health, Baltimore, Maryland, USA; University of Arizona

**Keywords:** dense granule protein, GRA, ESCRT, membrane transport, bradyzoite, *Toxoplasma*, cell membranes

## Abstract

The intracellular parasite Toxoplasma gondii adapts to diverse host cell environments within a replicative compartment that is heavily decorated by secreted proteins. In an attempt to identify novel parasite secreted proteins that influence host cell activity, we identified and characterized a transmembrane dense granule protein dubbed GRA64 (TGME49_202620). We found that GRA64 is on the parasitophorous vacuolar membrane (PVM) and is partially exposed to the host cell cytoplasm in both tachyzoite and bradyzoite parasitophorous vacuoles. Using co-immunoprecipitation and proximity-based biotinylation approaches, we demonstrated that GRA64 appears to interact with components of the host endosomal sorting complexes required for transport (ESCRT). Genetic disruption of GRA64 does not affect acute *Toxoplasma* virulence or encystation in mice, as observed via tissue cyst burdens in mice during chronic infection. However, ultrastructural analysis of Δ*gra64* tissue cysts using electron tomography revealed enlarged vesicular structures underneath the cyst membrane, suggesting a role for GRA64 in organizing the recruitment of ESCRT proteins and subsequent intracystic vesicle formation. This study uncovers a novel host-parasite interaction that contributes to an emerging paradigm in which specific host ESCRT proteins are recruited to the limiting membranes (PVMs) of tachyzoite and bradyzoite vacuoles formed during acute and chronic *Toxoplasma* infection.

## INTRODUCTION

The intracellular parasite Toxoplasma gondii is a widespread pathogen estimated to infect approximately one-third of human beings worldwide (1). The extensive prevalence of *Toxoplasma* can be partially attributed to its flexible life cycle, in which a wide variety of hosts can be infected, most commonly by ingesting material containing parasites within cystic structures (2, 3). Within intermediate hosts (where asexual replication occurs), T. gondii interconverts between two life stages that typify acute and chronic infection (4). The tachyzoite life stage spreads throughout the body of the host during acute infection and causes disease through host cell lysis and subsequent tissue damage, whereas the bradyzoite life-stage exhibits a quasi-dormant existence within intracellular tissue cysts, predominately in the brain and muscle tissue, during chronic infection (5). Bradyzoite differentiation is induced by various factors that can be summarized as stressors to the parasite which trigger a bradyzoite transcriptional program and an altered pattern of intracellular replication (6, 7). The absence of stressors, as might occur in the setting of compromised host immunity, is a permissive state that allows for bradyzoite reconversion back to the tachyzoite stage, leading to life-threatening encephalitis and other complications (8, 9). Because there are currently no treatments available which eradicate tissue cysts from chronically infected hosts, there is a major need to understand the processes that contribute to parasite latency.

Both the tachyzoite and bradyzoite life stages replicate within specialized intracellular vacuoles, termed either the parasitophorous vacuole (during tachyzoite infection) or the tissue cyst (during bradyzoite infection) (4). Parasitophorous vacuoles and tissue cysts are extensively modified by the parasites within them through the secretion of lipids and proteins into the vacuolar space and cyst matrix (10, 11). For example, the secretion of multi-lamellated vesicles from the basal ends of tachyzoites gives rise to the intravacuolar network (IVN) (12), which is known to play a pivotal role in the acquisition of host cell resources (13, 14). A seemingly analogous structure, the intracystic network, has been described in tissue cysts as well (11). Historically, the defining feature of tissue cysts has been the cyst wall, which appears as an electron dense conglomeration of vesicular and filamentous material that underlies the cyst membrane (11). Despite ultrastructural characterization of the tachyzoite parasitophorous vacuole and bradyzoite tissue cysts, much remains to be discovered regarding the number and functions of the proteins secreted into these specialized vacuoles and their contributions to parasitism. Many of the proteins found either in a soluble or insoluble membrane-associated state arise from secretory organelles called dense granules (15), which constitutively release dense granule proteins (GRAs) throughout intracellular development (16). Many GRA proteins do not contain domains with known functions, suggesting they might mediate novel processes unique to the parasite’s intracellular niche.

Despite their enigmatic nature, very few GRA proteins have had their functions in vesicle trafficking and nutrient acquisition elucidated. GRA2 and GRA6 are pivotal in forming the IVN during the early stages of tachyzoite parasitophorous vacuole development (17). GRA7 sequesters host endocytic organelles to the parasitophorous vacuole membrane (PVM) (18). GRA3 recruits the host Golgi and aids in the trafficking of Golgi vesicles across the PVM (19), and MAF1 recruits and tethers host mitochondria to the PVM (20). GRA17 and GRA23 have been shown to traffic small molecules across the PVM (21), and it is suspected that GRA17 serves the same purpose during bradyzoite development (22). Recently, an interaction between GRA14 and the host endosomal sorting complex required for transport (ESCRT) machinery was discovered, which mediates ESCRT-dependent virus-like particle budding and internalization of host cytosolic proteins at the PVM (23). The ESCRT machinery is comprised of ESCRT-0, ESCRT-I, ESCRT-II, ESCRT-III, the Vps4 complex, and associated accessory proteins (24), which sequentially interact to drive a wide variety of cellular processes such as membrane repair (25), membrane scission (26), phagophore closure (27), cytokinesis (28), membrane budding (29), and vesicle formation in multivesicular bodies (30). Several GRA proteins have been shown to be partially exposed to the host cell following their transmembrane insertion into the PVM, such as GRA5 (31), GRA6 (32), and GRA14 (33). GRA6 has been shown to influence host cell nuclear factor of activated T cells (NFAT) activity (34), while a specific GRA15 gene variant has been shown to induce host cell NF-κB activity (35), presumably through host cell exposure of GRA15 at the PVM. Although GRA15 is the main regulator of NF-κB, GRA7 and GRA14 also modify the nuclear localization of RelA/p65, a member of the NF-κB complex (36). Some GRA proteins have even been found to operate beyond the PVM interface, entering the host cell cytoplasm and nucleus and influencing host cell function by interacting with specific host cell proteins (37, 38).

In an effort to identify novel secreted *Toxoplasma* proteins that may directly influence host cell activity, we devised an *in silico* screen to predict genes encoding proteins with properties similar to those of known exported effector proteins. We identified one protein from this screen which was secreted into the vacuole, but not beyond the PVM or cyst membrane, likely due to the presence of a transmembrane domain. We set about characterizing this novel protein, TGME49_202620 (dubbed GRA64), in detail and found that its N terminus is exposed to the host cell during intracellular infection. Co-immunoprecipitation and proximity-based biotinylation approaches revealed that the most frequent host cell-interacting partners of GRA64 are ESCRT proteins, which canonically generate intraluminal vesicles away from the cytosol following their stepwise recruitment. Genetic deletion of GRA64 did not impair tachyzoite growth, nor did it impact cyst burden during chronic infection. However, ultrastructural analysis of Δ*gra64* tissue cysts demonstrated an enlargement of intraluminal vesicles adjacent to cyst membranes, which appeared to arise more frequently than in wild-type tissue cysts. Electron tomography of Δ*gra64* tissue cysts suggest these enlarged intraluminal vesicles to be cyst membrane invaginations, potentially indicating perturbed ESCRT-mediated scission events at the cyst membrane in the absence of GRA64. However, unlike GRA14 (23), GRA64 neither participates in ESCRT-dependent virus-like particle budding nor regulates ingestion of host cytosolic proteins. Altogether, our findings suggest that GRA64 is one of several membrane-bound GRA proteins facing the host cell that recruits the host ESCRT, the consequences of which have yet to be fully understood.

## RESULTS

### Discovery of a novel GRA protein, GRA64.

**FIG 1:**
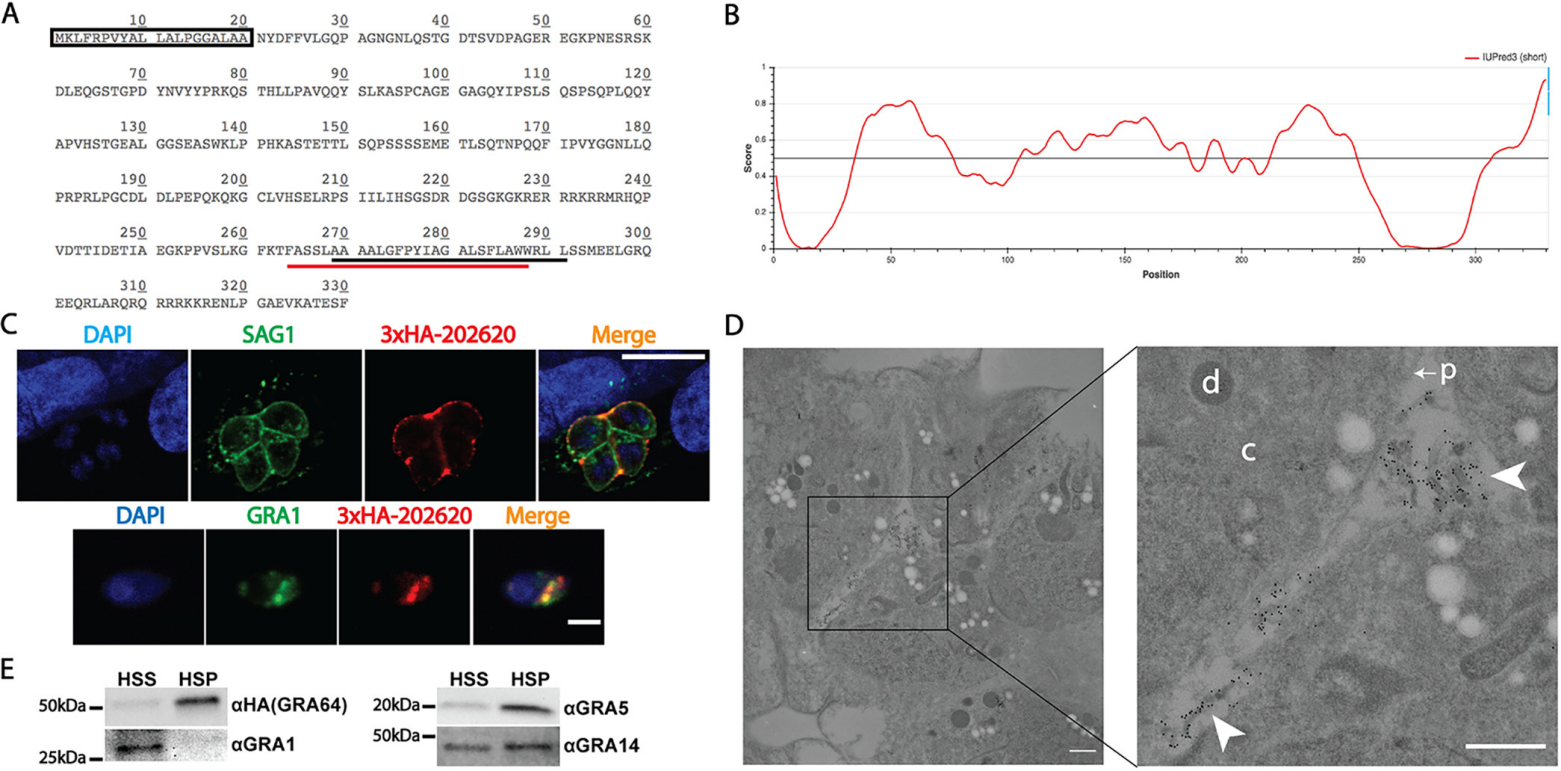
GRA64 is a membrane-bound dense granule protein localizing to the intravacuolar network during tachyzoite infection. (A) Amino acid sequence of *TGME49_202620*. The predicted signal peptide is indicated by a box (SignalP 5.0 prediction), while the predicted transmembrane domains are indicated by underlines (black, TMHMM prediction; red, Phobius prediction). (B) Results from IUPred3 short disorder prediction of TGME49_202620 protein intrinsic disorder are shown using medium smoothing (an approach within IUPred3 used to reduce the noise of amino acid residue free energy predictions based on the predictions of neighboring residues). Score indicates regions of low and high disorder. Low predicted disorder correlates with the signal peptide and transmembrane domain regions of the amino acid sequence indicated on the graph. (C) Top panel: IFA image of N terminus 3×HA epitope-tagged TGME49_202620 protein from a tachyzoite vacuole 1 day postinfection. TGME49_202620 is detected within the parasitophorous vacuole outside the parasite boundaries (ascertained by SAG1 labeling), with partial labeling of the parasitophorous vacuolar membrane (PVM). Scale bar = 10 μm. Bottom panel: immunofluorescence assay (IFA) images of an extracellular parasite expressing N terminus 3×HA epitope-tagged TGME49_202620 protein. TGME49_202620 (here, GRA64) colocalizes with GRA1. Scale bar = 5 μm. (D) Immunoelectron microscopy image of a tachyzoite vacuole expressing N terminus 3×HA epitope-tagged GRA64 protein, with select compartments labeled in the higher magnification inset image as follows: “d,” parasite dense granule; “c,” parasite cytoplasm; “p” with arrow, parasite plasma membrane; arrowheads, gold particle-labeled tubular material within the lumen of the parasitophorous vacuole. The 10-nm gold particles conjugated to anti-HA antibody appear to label the membranous structures of the intravacuolar network (IVN) within the parasitophorous vacuole lumen. The parasitophorous vacuole membrane was likely compromised by Triton X-100, which was used to permeabilize membranes for the labeling of GRA64 in this experiment. Bars, 500 nm. (E) Immunoblot images of GRA64, GRA1, GRA5, and GRA14 probed in high-speed supernatant (HSS, soluble) and high-speed pellet (HSP, membranous) fractions. GRA1, a soluble protein, is detectable only in the HSS fraction, whereas GRA64 is detectable predominately in the HSP fraction, similar to the previously described membrane-bound proteins GRA5 and GRA14.

The gene *TGME49_202620* was identified from an *in silico* screen aimed at characterizing novel exported parasite effector proteins by predicting the presence of the following molecular features in a given protein sequence: a signal peptide, a nuclear localization signal, and protein intrinsic disorder of greater than 70% (based on the predicted extent of disorder exhibited by known parasite-exported proteins). *TGME49_202620* is predicted to encode a protein containing a signal peptide and a transmembrane domain proximal to the C terminus (Fig. 1A). As assessed by the webserver IUPred3 (39), the gene product is predicted to contain regions of protein intrinsic disorder (Fig. 1B), suggesting a capability for promiscuous protein-protein interactions. The more ordered regions predicted by IUPred3 correspond to the signal peptide and transmembrane domain of TGME49_202620 (Fig. 1B). To confirm TGME49_202620 secretion and eventually generate a knockout strain, Type II PruΔ*ku80*Δ*hxgprt* (PruQ) parasites were endogenously tagged with three copies of the hemagglutinin tag (3×HA) appended to the N terminus of TGME49_202620, downstream of the predicted signal peptide. Immunofluorescence assays (IFAs) of N terminus-tagged TGME49_202620 protein demonstrated secretion into the lumen of the parasitophorous vacuole and partial PVM localization under tachyzoite growth conditions (Fig. 1C, top panel). IFAs of extracellular parasites from the endogenously tagged strain revealed that the TGME49_202620 protein co-localizes with the dense granule marker GRA1, indicating that this protein is likely packaged into dense granules prior to secretion into the parasitophorous vacuole (Fig. 1C, bottom panel). Given this result, we here refer to the *TGME49_202620* gene product as GRA64, and we used the endogenously tagged 3×HA strain for most of the subsequent experiments, unless otherwise specified. To assess the localization of GRA64 more accurately during intracellular infection, we performed immunoelectron microscopy of tachyzoite vacuoles. The images demonstrated that GRA64 signal is most frequently detected in association with membranous tubular structures reminiscent of the intravacuolar network (Fig. 1D). No PVM labeling was observed in this experiment, probably because the integrity of the PVM appeared to be compromised during the preparation of these samples due to the use of Triton X-100; however, no other detergent conditions were evaluated in these immunoelectron microscopy experiments.

To determine whether the predicted transmembrane domain conferred membrane-interacting properties to GRA64, we performed cellular fractionation experiments using material from infected monolayers containing tachyzoites (Fig. S1A illustrates a schematic of the fractionation procedure). Lysates were obtained by mechanically disrupting infected monolayers in PBS with a cell scrapper, passing the lysate through a 27G needle multiple times, and subjecting the lysate to low-speed centrifugation to remove intact parasites from the lysate. High-speed centrifugation was performed on the resulting low speed supernatant to separate soluble components (high-speed supernatant, HSS) from a membrane-enriched fraction (high-speed pellet, HSP). As expected, the soluble parasite antigen GRA1 was predominately detectable in the HSS fraction and undetectable in the HSP fraction, whereas the membrane-bound parasite antigens GRA5 and GRA14 were readily detectable in the HSP fraction (Fig. 1E). GRA64 exhibited a fractionation pattern similar to that of GRA5 (predominately in the HSP, Fig. 1E), indicating that GRA64 exhibits membrane-interacting properties. To assess whether GRA64 behaves as a peripheral or integral membrane protein, HSP fractions were subjected to treatments with various solutions and fractionated by high-speed centrifugation post-treatment. Whereas exposure to phosphate-buffered saline (PBS) or 1 M NaCl did not appreciably solubilize GRA64 from the HSP fraction, treatments with carbonate (0.1 M Na_2_CO_3_), 6 M urea, and the nonionic detergents NP-40 (1%) and Triton X-100 (1%) were capable of partially solubilizing GRA64 protein from the HSP fraction (Fig. S1B). As a membrane protein control, GRA14 was also probed for in these samples, and it was found to be robustly solubilized by both nonionic detergents but only partially solubilized by carbonate and urea (Fig. S1B). GRA14 is known to be partially solubilized by carbonate within dense granules, where it is thought to behave as an aggregated hydrophobic protein (33). The partial solubilization of GRA14 by carbonate and urea in this experiment suggests the presence of aggregated GRA proteins in the HSP fraction, in addition to membrane-inserted GRA proteins solubilized by detergent. Overall, the comparable fractionation pattern between GRA14 and GRA64 post-HSP treatment suggests that GRA64 behaves as an integral membrane protein following dense granule secretion, similar to what has been described for other GRA proteins with transmembrane domains, such as GRA5 and GRA14 (31, 33).

10.1128/mbio.01442-22.3 FIG S1 GRA64 behaves as a membrane-bound protein following cell fractionation. (A) Schematic illustrating the membrane fractionation approach and the generation of various fractions assessed as shown in Fig. 1E and this figure. (B) Immunoblots of high-speed pellet (HSP, “P”) and high-speed supernatant (“S”) fractions obtained after treating HSP fractions with either phosphate-buffered saline (PBS), 1 M NaCl, 0.1 M Na_2_CO_3_ (pH 11), 6 M Urea, 1% NP-40, or 1% Triton X-100 (TX-100). GRA64 is partially solubilized by carbonate, urea, and nonionic detergents, but not by PBS or 1 M NaCl (top panel). GRA14 appears to be partially solubilized by carbonate and urea, whereas NP-40 and TX-100 robustly solubilized GRA14 (bottom panel). Download FIG S1, TIF file, 1.0 MB. Copyright © 2022 Mayoral et al. 2022 Mayoral et al. https://creativecommons.org/licenses/by/4.0/ This content is distributed under the terms of the Creative Commons Attribution 4.0 International license.

Immunoblots of GRA64 reveal migration close to 50 kDa (Fig. 1E and Fig. S1B), which is slightly slower than the predicted size of ~39 kDa. GRA64 has a predicted *N*-glycosylation site at amino acid 55 and several phosphorylation sites detected in prior proteomic data sets deposited onto the *Toxoplasma* database ToxoDB (40), potentially accounting for the slower migration observed together with the intrinsically disordered structure predicted by IUPred3. No differences were observed in GRA64 migration from protein harvested from extracellular parasites, a 24-h infected tachyzoite culture, or a 3-day induced bradyzoite culture (data not shown).

### N terminus of GRA64 is exposed to the host cell cytoplasm during intracellular infection.

**FIG 2:**
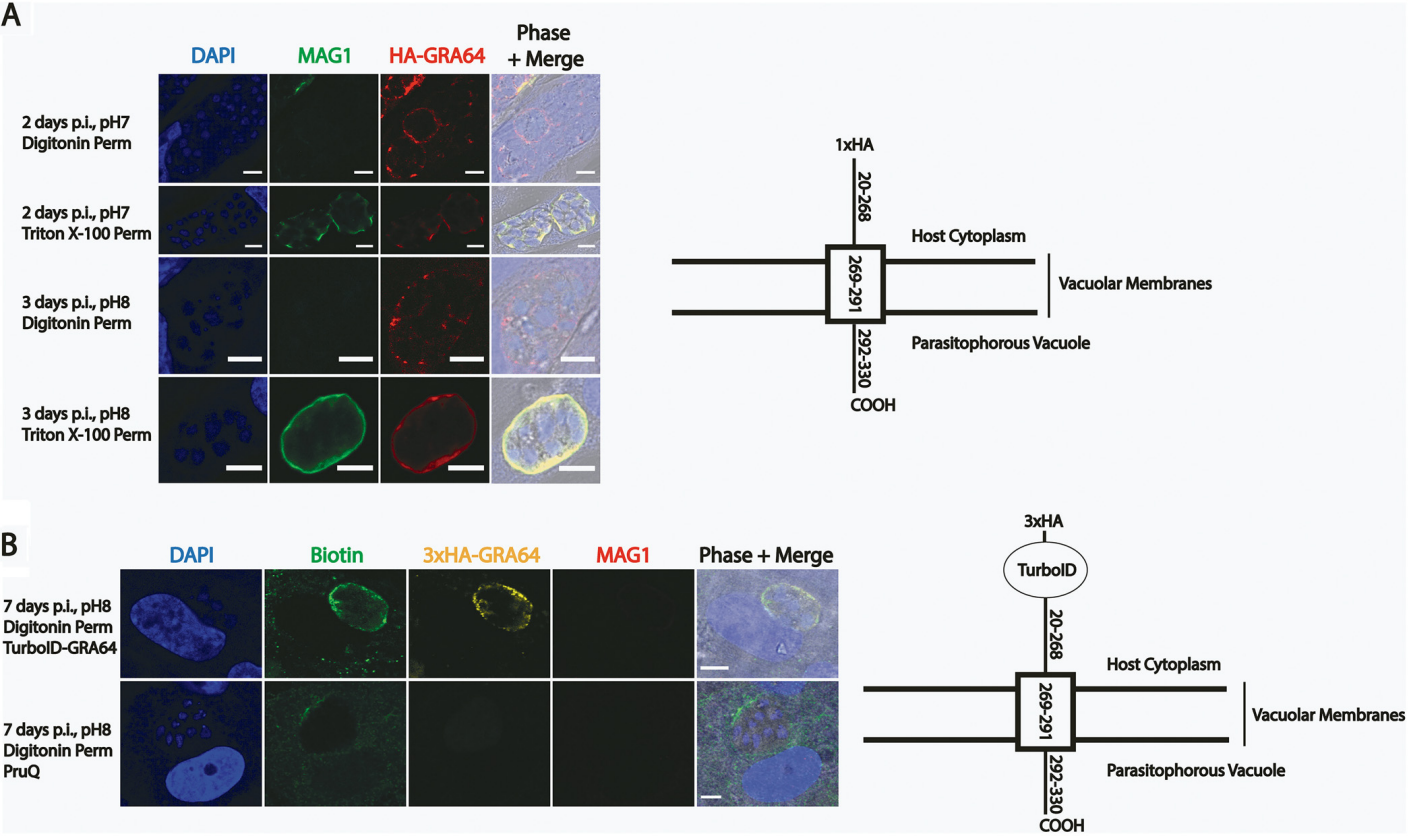
Epitope-tagged and TurboID-tagged GRA64 proteins are exposed to the host cell cytoplasm during intracellular infection. (A) IFA panels of parasites expressing GRA64 tagged at the N terminus with a 1xHA tag in tachyzoite vacuoles (2 days postinfection [dpi], pH 7) or induced bradyzoite vacuoles (3 dpi, pH 8). The digitonin concentration used for permeabilization in these experiments (0.001%) selectively permeabilizes the host cell membrane, but not the PVM. In contrast, 0.2% Triton X-100 fully permeabilizes the host cell and the PVM, as demonstrated by positive staining of MAG1, a parasite protein localizing to the lumen of parasitophorous vacuoles and to the cyst wall in differentiating parasite vacuoles. Scale bars = 5 μm. (B) IFA panels of parasites expressing GRA64 tagged at the N terminus with a 3×HA tag and the proximity-based biotinylating enzyme TurboID tag or untagged PruQ parasites in induced bradyzoite vacuoles (7 dpi, pH 8), supplemented with 150 μM biotin during the last 3 days of culture. Infected cells were permeabilized with digitonin (0.001%) to selectively permeabilizes the host cell membrane, but not the cyst membrane. Vacuoles were stained with αbiotin and αHA to confirm activity of the TurboID tag and expression of GRA64 via the 3×HA tag at the host cytoplasm interface, respectively. A lack of MAG1 labeling was used as an indicator for selective permeabilization. Scale bars = 5 μm.

We hypothesized that GRA64 is exposed to the host cell cytoplasm following insertion of the transmembrane domain into the lipid membranes of the PVM or IVN. Parasites expressing an N-terminal 1×HA-tagged version of GRA64 at the endogenous locus were used in selective permeabilization IFA experiments instead of the 3×HA endogenously tagged strain to minimize any potential interference by the HA tag in the membrane insertion process (Fig. 2A schematic). At a digitonin concentration of 0.001% (wt/vol) to selectively permeabilize the host cell plasma membrane, but not the PVM, to antibodies, GRA64 signal was detected in intact parasitophorous vacuoles, as assessed by the lack of labeling of MAG1, an abundant vacuolar protein (Fig. 2A, Digitonin Perm panels). GRA64 exposure to the host cell cytoplasm was detected both under tachyzoite (pH 7) and bradyzoite (pH 8) growth conditions (Fig. 2A). As expected, full permeabilization of host cell and parasitophorous vacuolar membranes with 0.2% Triton X-100 revealed labeling of both GRA64 and MAG1 (Fig. 2A, Triton X-100 Perm panels).

With the aim of eventually identifying host cell proteins which potentially interact with GRA64, we engineered parasites expressing a TurboID-GRA64 fusion protein. The proximity-based biotinylating enzyme TurboID (41) and a 3×HA epitope tag were appended to the N terminus of endogenous GRA64 (Fig. 2B, schematic). To assess whether the addition of the 3×HA-TurboID fusion tag might interfere with the membrane insertion and topology of the GRA64 protein, selective permeabilization experiments were again performed with this parasite strain under bradyzoite growth conditions for 7 days, supplemented with 150 μM exogenous biotin during the final 3 days of culture. TurboID-GRA64 fusion protein was also detected in intact parasitophorous vacuoles, as determined by the detection of 3×HA signal in vacuoles without MAG1 signal (Fig. 2B). Biotinylation was also detected in intact parasitophorous vacuoles using anti-biotin antibody, indicating that the TurboID enzyme is also active specifically at the host cell-exposed vacuolar membrane interface. Hence, the N-terminal portion (with respect to the predicted transmembrane domain) of various GRA64 fusion proteins appears to be consistently exposed to the host cell cytoplasm. Although TurboID-mediated biotinylation has been demonstrated to occur with shorter labeling times, we did not detect significant biotin signals by IFA at the host cytoplasm-cyst membrane interface of intact TurboID-GRA64 vacuoles in which supplemental biotin was only provided for 1 day prior to fixation. Hence, we opted for labeling times of 3 days in subsequent experiments to allow for the accumulation of biotinylated host proteins proximal to the TurboID-GRA64 fusion protein at the PVM interface, thus maximizing the likelihood of labeling and detecting host proteins.

### GRA64 interacts with host cell proteins from the endosomal sorting complexes required for transport.

Given the host cell exposure of GRA64, we hypothesized that certain host cell proteins interact with GRA64. Co-immunoprecipitations (Co-IPs) were performed using protein lysates from tachyzoite-infected human fibroblast cultures, using PruQ parasites expressing endogenously tagged 3×HA-GRA64 protein (24 h postinfection). Co-IPs of GRA64 in protein lysates from bradyzoite-infected human fibroblasts (4 days postinfection [dpi]) and from mouse primary cortical neuron infected cultures (2 dpi) were also performed to determine if any host cell protein associations were common between different life stages, infection time points, host cell types, and host species. Untagged PruQ parasites cultured under the same conditions were used as a negative control for all experiments to identify proteins that non-specifically bound to the anti-HA antibody-coated magnetic beads. Two independent experiments were performed for each condition. Following overnight incubation of harvested proteins with anti-HA magnetic beads, the beads were washed, proteins were eluted with Laemmli buffer, detergent was removed, and the proteins were digested into peptides in S-TRAP columns and subsequently analyzed by liquid chromatography with tandem mass spectrometry (LC-MS/MS).

**FIG 3:**
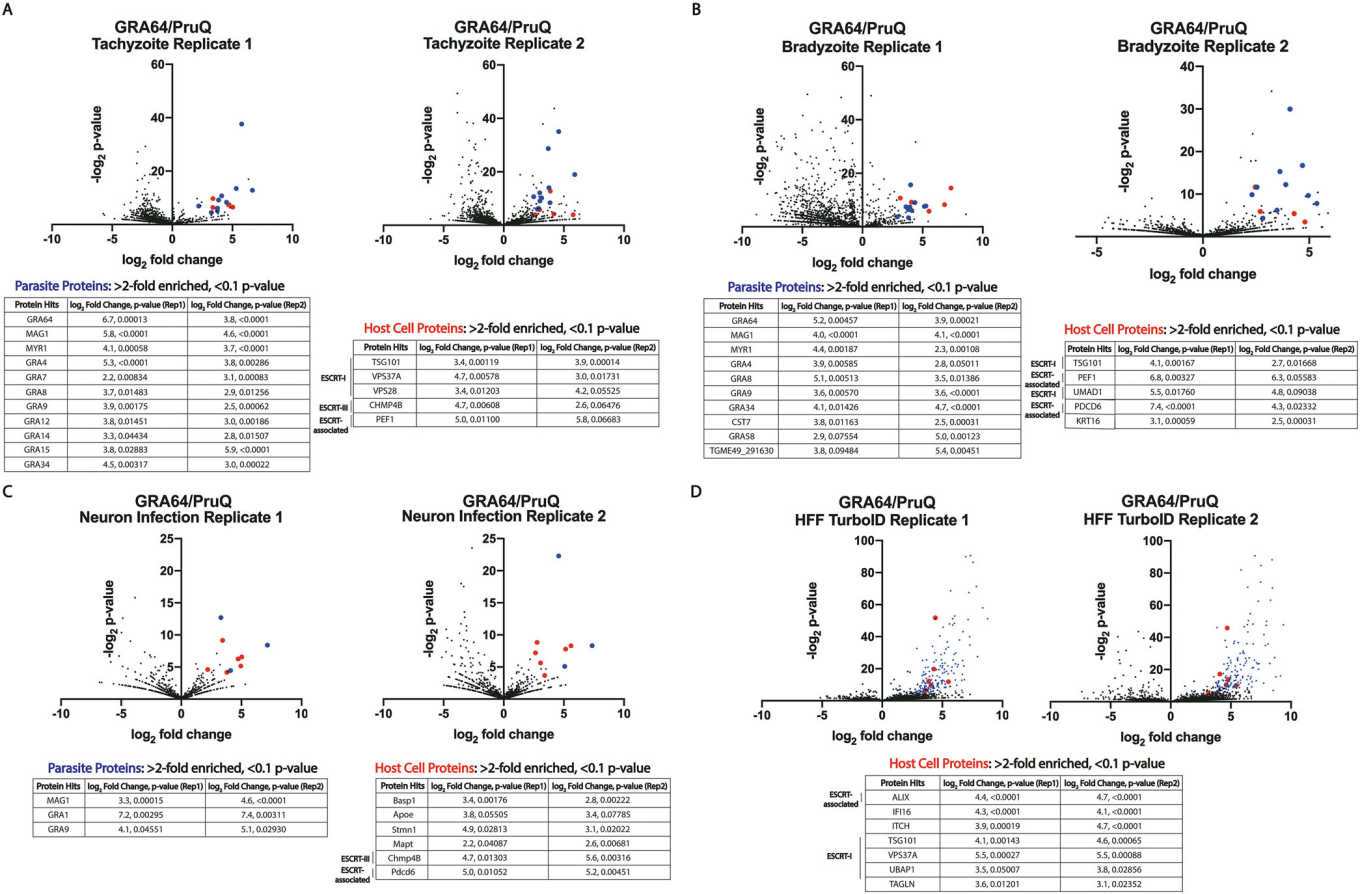
GRA64 interacts with host endosomal sorting complexes required for transport (ESCRT) proteins. Volcano plots of liquid chromatography with tandem mass spectrometry (LC-MS/MS) results from co-immunoprecipitations (Co-IP) of endogenously tagged 3×HA-GRA64 protein from either (A and B) infected human fibroblast monolayers under (A) tachyzoite or (B) bradyzoite growth conditions (1 and 4 dpi, respectively), or from (C) mouse primary cortical neuron cultures (2 dpi). In each volcano plot, red and blue data points indicate a host or parasite protein, respectively, classified as significant in two independent experiments (log_2_-fold enrichment ≥ 2 and *P*  < 0.1 in both replicate 1 and replicate 2). Under each condition, the host proteins identified as significant hits were largely ESCRT or ESCRT-associated proteins. (D) TurboID-GRA64 expressing parasites were used to identify proximal proteins in human foreskin fibroblast cultures. Lists of all host cell proteins (red dots in volcano plots) with log_2_-fold enrichment ≥ 2 and *P*  ≤ 0.1 are listed in the tables below each figure panel. Significantly enriched parasite protein hits (blue dots) are provided in Supplementary Data Set S2.

Among the parasite proteins significantly enriched from tachyzoite infected fibroblast samples (log_2_-fold enrichment ≥ 2.0, *P* value ≤ 0.10 in two independent experiments), various GRA proteins were identified, as expected based on the localization of GRA64 in the vacuole and its presence in dense granules within the parasite (Fig. 1C). The only host cell proteins significantly enriched from tachyzoite-infected fibroblast samples were various proteins belonging to or associated with the endosomal sorting complexes required for transport (ESCRT) (Fig. 3). Specifically, representatives from the ESCRT-I (TSG101, VPS37A, VPS28) and ESCRT-III (CHMP4B) complexes were enriched in most Co-IP experiments (Fig. 3A to C), as well as proteins associated with ESCRT recruitment (PDCD6 and UMAD1). However, it is worth noting that GRA64 was not detected as being significantly enriched in the neuron Co-IPs (Fig. 3C), likely due to the low protein enrichment overall from neuron infected cultures, resulting in greater variability in the LC-MS/MS analysis pipeline. Despite the absence of significant bait-protein enrichment (i.e., GRA64) in the neuron experiment, the data demonstrate that parasite and host cell proteins similar to those found from fibroblast cultures were significantly enriched (GRA and ESCRT proteins), indicating an intriguing common recruitment of host proteins at the IVN/PVM interface (and analogous structures in tissue cysts) across each condition tested.

The Co-IP approach necessitates the lysis of host cells to immunoprecipitate GRA64 protein from the parasitophorous vacuole, which may lead to artificial interactions that do not normally occur during infection. To determine whether ESCRT protein proximity to GRA64 could be detected in living cells, we utilized proximity-based biotinylation with TurboID, using the afore-mentioned TurboID-GRA64-expressing parasite strain (Fig. 2B) and an untagged parasite strain as a control. Streptavidin resin was used to enrich biotinylated proteins in two independent experiments, harvesting proteins from bradyzoite-induced cultures supplemented with exogenous biotin 3 days postinfection. The results demonstrated that three ESCRT-I proteins (TSG101, VPS37A, UBAP1) and the accessory ESCRT protein ALIX were identified as significantly enriched (log_2_-fold enrichment ≥ 2.0, *P* value ≤ 0.1) in TurboID-GRA64 cultures compared to that in untagged cultures grown under similar conditions in both independent experiments (Fig. 3D). Hence, both Co-IP and TurboID approaches provide evidence for an association between GRA64 and host ESCRT proteins. TurboID labeling experiments were also performed during infection in mouse primary cortical neurons. No host proteins were found to be significantly enriched in TurboID-labeled samples in two independent experiments with neurons, despite evidence for successful TurboID labeling based on the significant enrichment of parasite proteins (Fig. S2). However, in both the Co-IP and TurboID experiments in which neurons were used as host cells, fewer peptides were detected overall by LC-MS/MS compared to that in the fibroblast experiments (Data Sets S1 and S2 in the supplemental material), suggesting that less total protein was harvested from neuron lysates than from fibroblast lysates.

10.1128/mbio.01442-22.4 FIG S2 TurboID-GRA64 liquid chromatography with tandem mass spectrometry (LC-MS/MS) results from infection of mouse primary cortical neuron cultures. TurboID-GRA64-expressing parasites were used to identify proximal proteins in infected mouse primary cortical neuron cultures in two independent experiments. A list of all parasite proteins (blue dots in the volcano plot) with log_2_-fold enrichment ≥ 2 and *P* ≤ 0.1 are listed in the table here, while the ESCRT and ESCRT-associated proteins identified as significantly enriched in other datasets (see Fig. 3) are also listed for comparison. Download FIG S2, TIF file, 2.5 MB. Copyright © 2022 Mayoral et al. 2022 Mayoral et al. https://creativecommons.org/licenses/by/4.0/ This content is distributed under the terms of the Creative Commons Attribution 4.0 International license.

**FIG 4:**
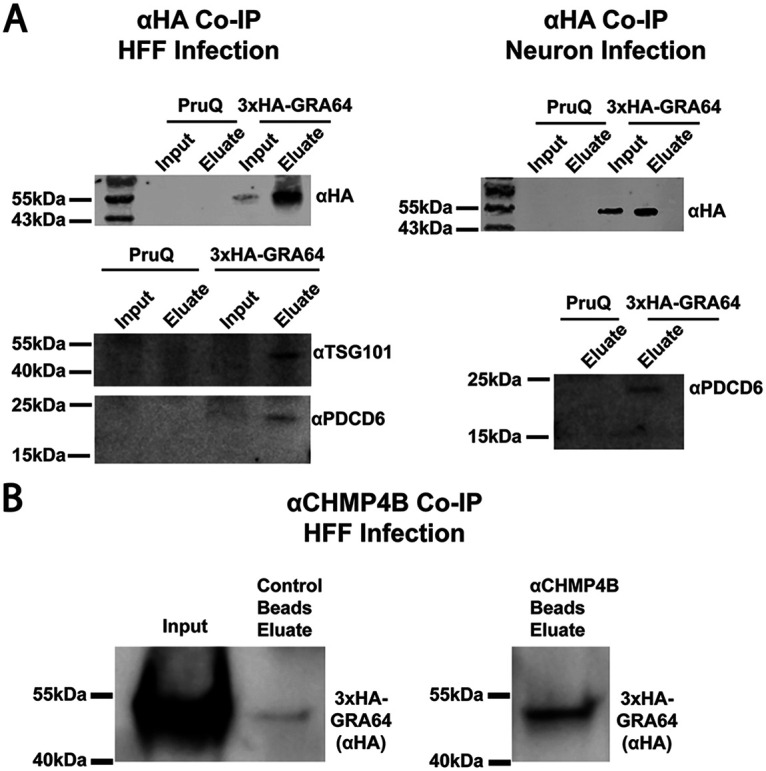
Immunoblot confirmation of ESCRT protein enrichment by GRA64. (A) Immunoblots of samples from an anti-HA Co-IP using either 3×HA-GRA64-tagged or untagged PruQ parasites as a control. Protein lysates were harvested for Co-IP from either human foreskin fibroblast infected monolayers (left panel) or from neuron infected cultures (right panel). Robust enrichment of GRA64 is seen in the eluate fraction compared to the input fraction (top left panel under “3×HA-GRA64”). Both TSG101 and PDCD6 were only detected in the 3×HA-GRA64 eluate samples and not in the control samples (bottom left panel), indicating ESCRT protein enrichment, in agreement with the LC-MS/MS results. Similarly, PDCD6 was only detected the 3×HA-GRA64 eluate and not in the control eluate from neuron infection samples (bottom right panel). (B) Immunoblot of 3×HA-GRA64 protein immunoprecipitated from dynabeads conjugated with anti-CHMP4B antibody (and unconjugated beads as a control). There is a notable enrichment of GRA64 using CHMP4B-conjugated beads compared to that when using unconjugated beads.

To help validate the results obtained by LC-MS/MS analysis, 3×HA-GRA64 pulldowns with anti-HA beads were repeated under tachyzoite growth conditions in human foreskin fibroblast cultures and during neuron infection (Fig. 4A). Clear enrichment of TSG101 and PDCD6 was observed in the human foreskin fibroblast infection eluates from 3×HA-GRA64 samples, but not in the untagged control eluates (Fig. 4A, left panel). Similarly, PDCD6 was found to be enriched by GRA64 pulldown during neuron infection (Fig. 4A, right panel), confirming the LC-MS/MS findings. A reciprocal Co-IP was also performed using antibody to the ESCRT-III protein CHMP4B, conjugated to magnetic dynabeads. As a control, unconjugated dynabeads were used in parallel. Incubating control and CHMP4B-conjugated magnetic beads with lysates from cultures infected by tachyzoites expressing 3×HA-tagged GRA64 protein demonstrated CHMP4B enrichment of GRA64 over control beads after several wash steps and elution, as determined by immunoblotting (Fig. 4B). These data suggest that the interaction between GRA64 and CHMP4B is reproducible, at least during the artificial Co-IP cell lysis and wash conditions used in this experiment. Altogether, LC-MS/MS results and pulldowns with GRA64 and CHMP4B demonstrate that GRA64 interacts with specific ESCRT-I, ESCRT-III, and accessory ESCRT proteins.

### Genetic disruption of GRA64 does not affect tachyzoite growth *in vitro*, virulence in mice, or cyst burden during chronic mouse infection.

To determine how GRA64 might contribute to parasite fitness, we next disrupted and complemented the GRA64 gene in PruQ parasites using a previously described Cas9 strategy (42). Immunoblots of protein lysates from each strain revealed, as expected, the absence of 3×HA-GRA64 protein in the Δ*gra64* strain and comparable amounts of GRA64 protein in the parental (3×HA-GRA64) and complemented (GRA64-COMP) strains (Fig. S3A). Neither *in vitro* growth in fibroblasts (determined by plaque assays, Fig. S3B) nor acute virulence *in vivo* (Fig. S3C) was affected in PruQΔ*gra64* parasites, indicating that GRA64 is dispensable for tachyzoite growth. We next assessed cyst burdens in CBA/J mice chronically infected with PruQ strain parasites 30 days postinfection. In two independent experiments, we observed a trend for reduced cyst burdens in mouse brains infected with the Δ*gra64* PruQ strain compared to those in mice infected with its parental and complemented counterparts (Fig. S4A). To further evaluate the cyst phenotype, in two independent experiments we quantified cyst burden in equivalent GRA64 strains generated in the relatively more cystogenic ME49Δ*ku80*Δ*hxgprt* (ME49Q) background. Immunoblot validation demonstrated comparable endogenous expression of 3×HA-tagged GRA64 protein between the parental and complemented strains, as well as the absence of 3×HA-GRA64 in the ME49QΔ*gra64* strain (Fig. S4B). Cyst burden results showed an overall increase in cyst yields, but no difference in ME49QΔ*gra64* cyst burdens compared to those of the complemented strain. Altogether, the data suggest that GRA64 does not significantly influence cyst burden in a predictable manner (Fig. S4C).

10.1128/mbio.01442-22.5 FIG S3 Δ*gra64* tachyzoites exhibit no growth defects *in vitro* or virulence defects *in vivo*. (A) Immunoblot demonstrating the absence of GRA64 protein expression in the PruQ knockout strain (Δ*gra64*) and comparable amounts of protein expressed in the PruQ parental (3×HA-GRA64) and complemented PruQ strain (GRA64-COMP). TgALD1 was used as a parasite-specific loading control. (B) Plaque assays in human fibroblasts monolayers cultures following 14 days of infection under tachyzoite growth conditions show no statistically significant difference in parasite growth, as measured by plaque sizes between each strain in three independent experiments. Representative images of plaques are provided for each strain beside the violin plots. (C) C57Bl/6 mouse mortality was recorded over a span of 30 days post-intraperitoneal infection, using 2,000 tachyzoites of each strain to infect 10 mice each. No significant differences in the survival curves were noted in this experiment (n.s., not significant). Download FIG S3, TIF file, 2.5 MB. Copyright © 2022 Mayoral et al. 2022 Mayoral et al. https://creativecommons.org/licenses/by/4.0/ This content is distributed under the terms of the Creative Commons Attribution 4.0 International license.

10.1128/mbio.01442-22.6 FIG S4 Disruption of GRA64 does not significantly affect tissue cyst burdens. (A and C) Cyst burdens were measured from the brains of chronically infected CBA/J mice (30 days postinfection) using (A) PruQ strain or (C) ME49Q strain parasites. (A) *P* values were calculated from a one-way analysis of variance (ANOVA) test for significance in replicate 1 and a Kruskal-Wallis test in replicate 2 (because data were not normally distributed in replicate 2). The data indicate a trend of fewer cysts formed during Δ*gra64* infection compared to that during infection with the parental and complement strains in both experiments. No significant differences in cyst burden between parental and complement strains were present in either experiment. (B) Immunoblot of 3×HA-GRA64 expression from the endogenous locus of various strains. ME49Q indicates the untagged strain used to generate the 3×HA-GRA64 “parental” strain, and hence no 3×HA-GRA64 protein was detected in the ME49Q protein lane. 3×HA-GRA64 protein is comparable between the parental (3×HA-GRA64) and complement (GRA64-COMP) strains, whereas it is absent in the ME49QΔ*gra64* knockout strain. SAG1 was used as a parasite loading control. (C) *P* values were calculated using a one-way ANOVA test for significance in both replicates. Data show higher cyst numbers using a more cystogenic strain; however, no significant differences in cysts formed during Δ*gra64* infection were noted compared to wild-type, parental, and complement strain infections in both experiments. Download FIG S4, TIF file, 2.2 MB. Copyright © 2022 Mayoral et al. 2022 Mayoral et al. https://creativecommons.org/licenses/by/4.0/ This content is distributed under the terms of the Creative Commons Attribution 4.0 International license.

### Tissue cysts formed by Δ*gra64* parasites demonstrate perturbed cyst ultrastructure.

**FIG 5:**
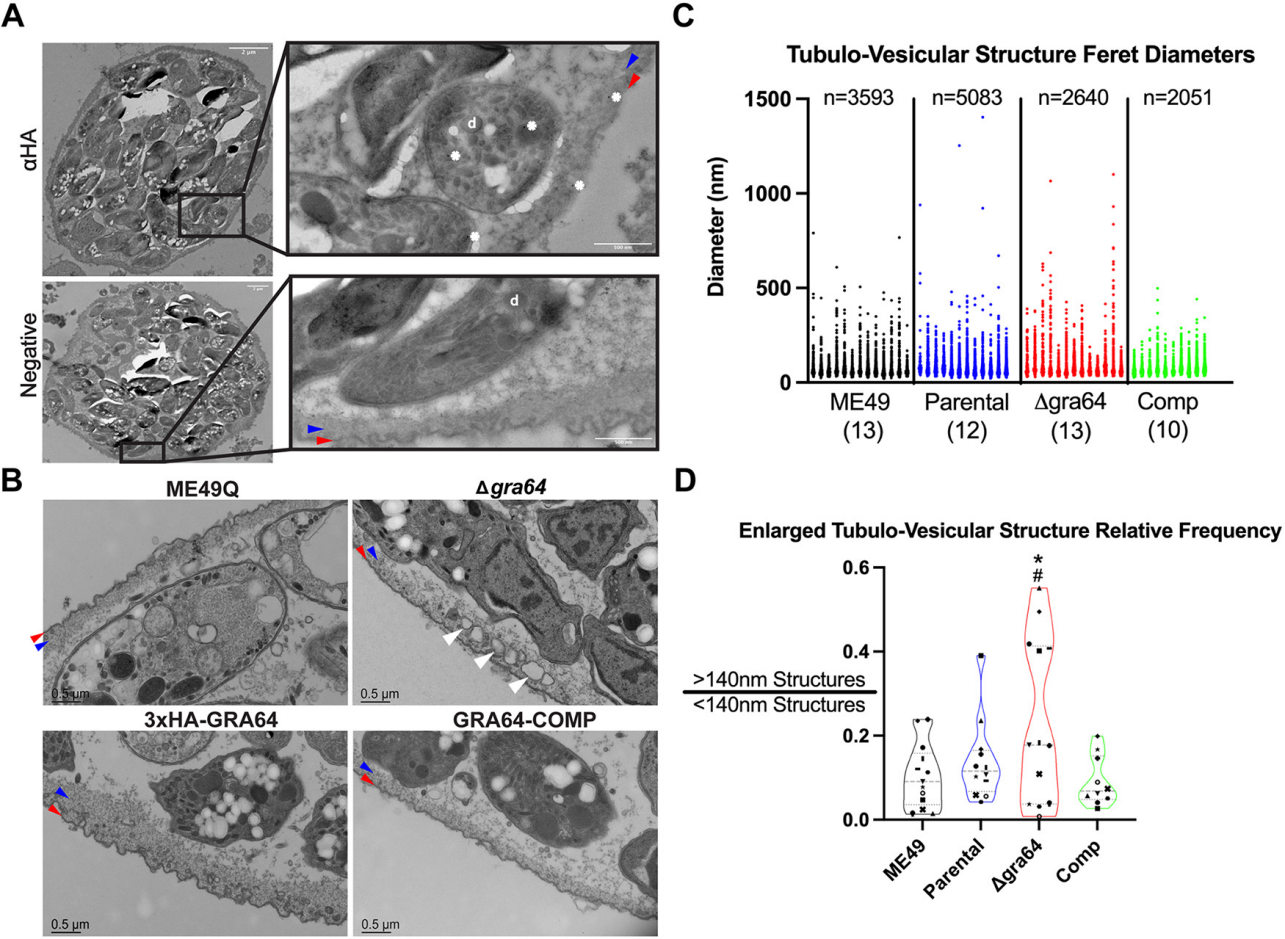
Δ*gra64*-derived tissue cysts exhibit a change in ultrastructure. (A) Immunogold labeling (using rat anti-HA and anti-rat 10-nm gold particle-conjugated antibodies) of GRA64-COMP strain tissue cysts demonstrates GRA64-positive signal (white asterisks) within dense granule (“d”) structures in bradyzoites and within the cyst wall. A GRA64-COMP tissue cyst labeled with only anti-rat gold-conjugated antibody is shiwn in the panel below for comparison (“negative”). Scale bars = 2 μm and 500 nm for magnified inserts. (B) Magnified views of tissue cyst walls and cyst membranes formed by each strain as indicated. Note the presence of large vesicular structures within the cyst and proximal to the cyst wall in the Δ*gra64* panel (white arrowheads). Scale bars = 0.5 μm. In panels A and B, red and blue arrowheads indicate the cyst membrane and the electron-dense cyst wall, respectively. (C) Graph plotting all Feret diameter measurements obtained for a particular cyst from either the ME49Q, parental (“3×HA-GRA64” in Panel B), Δ*gra64*, or complemented (Comp) strain. Each column of data points indicates measurements obtained from a single cyst (ranging from 78 to 887 measurements). Sample size above each group indicates the total number of measurements made for each strain; numbers in parentheses underneath each strain indicate the number of cysts analyzed per strain. Feret diameter: the longest distance between two points of a tubulo-vesicular structure tracing. (D) Violin plots demonstrating the relative frequency of enlarged tubulo-vesicular structures using a criterion of >140-nm diameter to define an enlarged tubulo-vesicular structure. Results from one-way analysis of variance (ANOVA) tests are only shown for comparisons in which *P* < 0.05: *, *P* < 0.05 between the Δ*gra64* and ME49Q strains; #, *P* < 0.05 between the Δ*gra64* and complemented strains. The number of cysts analyzed for each strain are shown in Fig. 5C. The symbols for each data point comprise a key indicating a specific cyst for each group; the same convention is used for all graphs in Fig. S6.

We next evaluated the localization of GRA64 in tissue cysts formed *in vivo*. Immunogold labeling of PruQ strain-complemented GRA64 cysts (obtained in sufficient numbers from chronic mouse infection experiments post-purification for examination by ImmunoEM) revealed labeling of the cyst wall region and parasite dense granules using anti-HA antibody, providing evidence that GRA64 is indeed expressed by mature bradyzoites *in vivo* (Fig. 5A). Furthermore, we assessed whether tissue cysts formed *in vivo* by Δ*gra64* parasites exhibited any morphological defects related to GRA64-ESCRT interaction. We hypothesized that defects in the recruitment of host ESCRT proteins at the cyst membrane interface could result in more prominent “stalled” cyst membrane invaginations due to the lack of efficient ESCRT-mediated membrane scission. Using the wild-type (ME49Q), GRA64 parental (3×HA-GRA64), GRA64 knockout (Δ*gra64*), and complemented (GRA64-COMP) ME49Q strains, we harvested and purified cysts by Percoll from mouse brains chronically infected for 4 weeks. Post-purification and fixation, cysts were analyzed by electron microscopy. We found that while wild-type, parental, and complemented cysts exhibited standard cyst wall architecture (electron dense material and small tubulo-vesicular material underneath the cyst membrane), Δ*gra64* cysts tended to exhibit large tubulo-vesicular structures within the cyst wall and proximal to the cyst membrane (Fig. 5B, Δ*gra64* panel, white arrowheads). To further investigate these large structures, we performed electron tomogram analysis of thicker sections (250 nm) from *in vivo* brain cysts of ME49Q (Movie S1) and Δ*gra64* (Movie S2) strains. The ME49Q tomogram demonstrates narrow cyst membrane invaginations with occasional vesicles trapped within the lumens of these invaginations. We further traced the vesicular structures in the Δ*gra64* tomogram to determine if they were continuous with the cyst membrane and observed a few close vesicular-cyst membrane contacts (Fig. S5). These large vesicular structures potentially represent enlarged invaginations that have not been efficiently excised from the cyst membrane due to perturbed ESCRT recruitment. However, we cannot conclude with certainty the nature of these seemingly aberrant structures from static images alone.

10.1128/mbio.01442-22.1 MOVIE S1 Electron tomogram of ME49Q tissue cyst. Note the vesicles and tubules in the cyst wall. The dots are gold 10-nm particles used for alignment during tomogram reconstruction. Download Movie S1, MPG file, 11.2 MB. Copyright © 2022 Mayoral et al. 2022 Mayoral et al. https://creativecommons.org/licenses/by/4.0/ This content is distributed under the terms of the Creative Commons Attribution 4.0 International license.

10.1128/mbio.01442-22.2 MOVIE S2 Electron tomogram of ME49QΔ*gra64* tissue cyst. Note the large vesicles adjacent to the cyst membrane and the lack of smaller vesicles and tubules in the cyst wall. The dots are gold 10-nm particles used for alignment during tomogram reconstruction. Download Movie S2, MPG file, 13.1 MB. Copyright © 2022 Mayoral et al. 2022 Mayoral et al. https://creativecommons.org/licenses/by/4.0/ This content is distributed under the terms of the Creative Commons Attribution 4.0 International license.

10.1128/mbio.01442-22.7 FIG S5 Disruption of GRA64 *in vivo* brain cyst exhibits large vesicular structures that have close contact with the cyst membrane. Cysts were purified from the brains of chronically infected CBA/J mice (30 days postinfection) using the (A and B) ME49Q and (C and D) ME49QΔ*gra64* parasites, prepared for EM serial imaging, and reconstructed. (A and C) Vesicles (shown in yellow, blue, green, and cyan), cyst wall (shown in magenta), and parasite membrane (shown in red) were traced using 3dmod within the IMOD software from the cyst shown in Movies S1 and S2. (B and D) Reconstruction model comprised of each serial image traced for the ME49Q and ME49QΔ*gra64* cysts. Download FIG S5, TIF file, 0.7 MB. Copyright © 2022 Mayoral et al. 2022 Mayoral et al. https://creativecommons.org/licenses/by/4.0/ This content is distributed under the terms of the Creative Commons Attribution 4.0 International license.

10.1128/mbio.01442-22.8 FIG S6 Additional electron microscopy measurements and analyses of tubulo-vesicular structures from *in vivo* ME49 strain tissue cysts. (A) Panel of violin plot graphs showing the average tubulo-vesicular densities, areas, diameters, and circularities of the ME49Q, parental, Δ*gra64,* and complement strains. Results from one-way ANOVA (used for the average area, diameter, and circularity analyses) or Kruskal-Wallis tests (used for the average tubulo-vesicular density analysis) are only shown for *P* values of <0.06. (B) Violin plots demonstrating the relative frequency of enlarged tubulo-vesicular structures, using a criterion of >100-nm diameter to define an enlarged tubulo-vesicular structure. Results from one-way ANOVA tests are only shown for *P* values of <0.06. (C) Panel of violin plot graphs showing the stratified average tubulo-vesicular areas, diameters, and circularities for each strain, using measurements obtained only from tubulo-vesicular structures of >140-nm in diameter. No statistically significant differences were noted between any strains using either one-way ANOVA or Kruskal-Wallis tests. For all graphs, the same symbol key as in Fig. 5D is used to indicate a particular cyst for a given strain. The sample size for each strain is identical for each graph: ME49, 13 cysts; parental, 11; Δ*gra64*, 13; complemented, 11. Download FIG S6, TIF file, 2.2 MB. Copyright © 2022 Mayoral et al. 2022 Mayoral et al. https://creativecommons.org/licenses/by/4.0/ This content is distributed under the terms of the Creative Commons Attribution 4.0 International license.

We next sought to quantify the average area, diameter, and circularity of the tubulo-vesicular structures within the cyst walls of each strain, as well as the frequencies of the enlarged tubulo-vesicular structures noted in Fig. 5B, hypothesizing that the Δ*gra64* strain would exhibit the greatest difference across each of these metrics compared to the ME49Q, parental, and complemented strains. A total of 684 thin-section EM images from 47 cysts were analyzed blindly by manually tracing tubulo-vesicular structures (TVs) within the cyst wall while also measuring their respective Feret diameters (i.e., the longest distance between two points of a TV tracing), areas, and circularity using ImageJ software. A total of 13,367 tracings were made from all the images, with individual measurements per cyst ranging from 78 to 887 tracings (Fig. 5C). We noted that the most measurements were made in the parental strain (Fig. 5C); intriguingly, the number of TVs measured per field of view was significantly greater in the parental strain compared to that in the knockout strain (Fig. S6A), although the biological significance of this result is unclear. The average diameter of TVs measured in all ME49Q cysts (mean = 77.14 nm, standard deviation [SD] = 56.31) is similar to the average diameter reported for cyst matrix vesicles in a prior detailed analysis of wild-type cyst morphology (mean = 72.56 nm, SD = 24.43) (11). Differences in the standard deviation between our results and those of the prior study may reflect the grouping of tubule, vesicle, and cyst membrane invagination tracings in our analysis. Notably, while the average diameters of TVs from pooled parental and complemented cysts are similar to the ME49Q average (mean = 81.60, SD = 58.33; and mean = 81.72, SD = 44.88, respectively), the average diameter measured from the Δ*gra64* strain is larger by comparison (mean = 94.91, SD = 83.88). The average diameter of TVs in the Δ*gra64* strain demonstrated an increasing trend compared to that in the ME49Q strain (Fig. S6A), whereas the average area of TVs was significantly greater in the Δ*gra64* strain compared to that in the ME49Q strain (Fig. S6A). The circularities of TVs were not significantly different between any strains (Fig. S6A).

We used a 140-nm diameter cutoff to define an “enlarged” TV, reasoning that most of the diameter measurements made among all strains fall below this value (Fig. 5C). This value is also the upper limit of cyst vesicle diameter ranges documented in the aforementioned cyst morphology study (11). Using this cutoff, we found that the relative frequency of enlarged TVs (the proportion of “enlarged” TVs to “normal” TVs, or >140-nm TVs/<140-nm TVs) was significantly greater in the Δ*gra64* strain than in the ME49Q and complemented strains, while only a trend is observed when comparing the Δ*gra64* strain to the parental strain (Fig. 5D). Similar results were obtained when using a less stringent definition for an enlarged TV (>100-nm diameter, Fig. S6B). When stratifying the data and only considering enlarged TVs per the >140-nm diameter criterion, no significant differences were observed in average TV area, diameter, or circularity between the strains (Fig. S6C). Altogether, these data indicate that the average area and average diameter of TVs, in addition to the average relative frequencies of enlarged TVs, are greatest in the Δ*gra64* strain compared to that in the other strains. In contrast, the average circularity of TVs is not significantly different between the strains, nor are the enlarged TV structures themselves significantly different in area, diameter, or circularity among the strains. We also note that the distribution of enlarged TV relative frequencies appears to be bimodal in the Δ*gra64* strain (Fig. 5D, Fig. S6B). We confirmed that the population exhibiting an enlarged TV relative frequency of >0.3 (Fig. 5D) does not exclusively arise from cysts obtained from a particular mouse experiment. Thus, it is currently unclear what factor(s) might influence the bimodality seen in the phenotype of Δ*gra64 in vivo* cysts harvested from mouse brain.

### GRA64 does not recruit ESCRT machinery in a virus-like particle assay and is not required for internalization of host cytosolic proteins.

**FIG 6:**
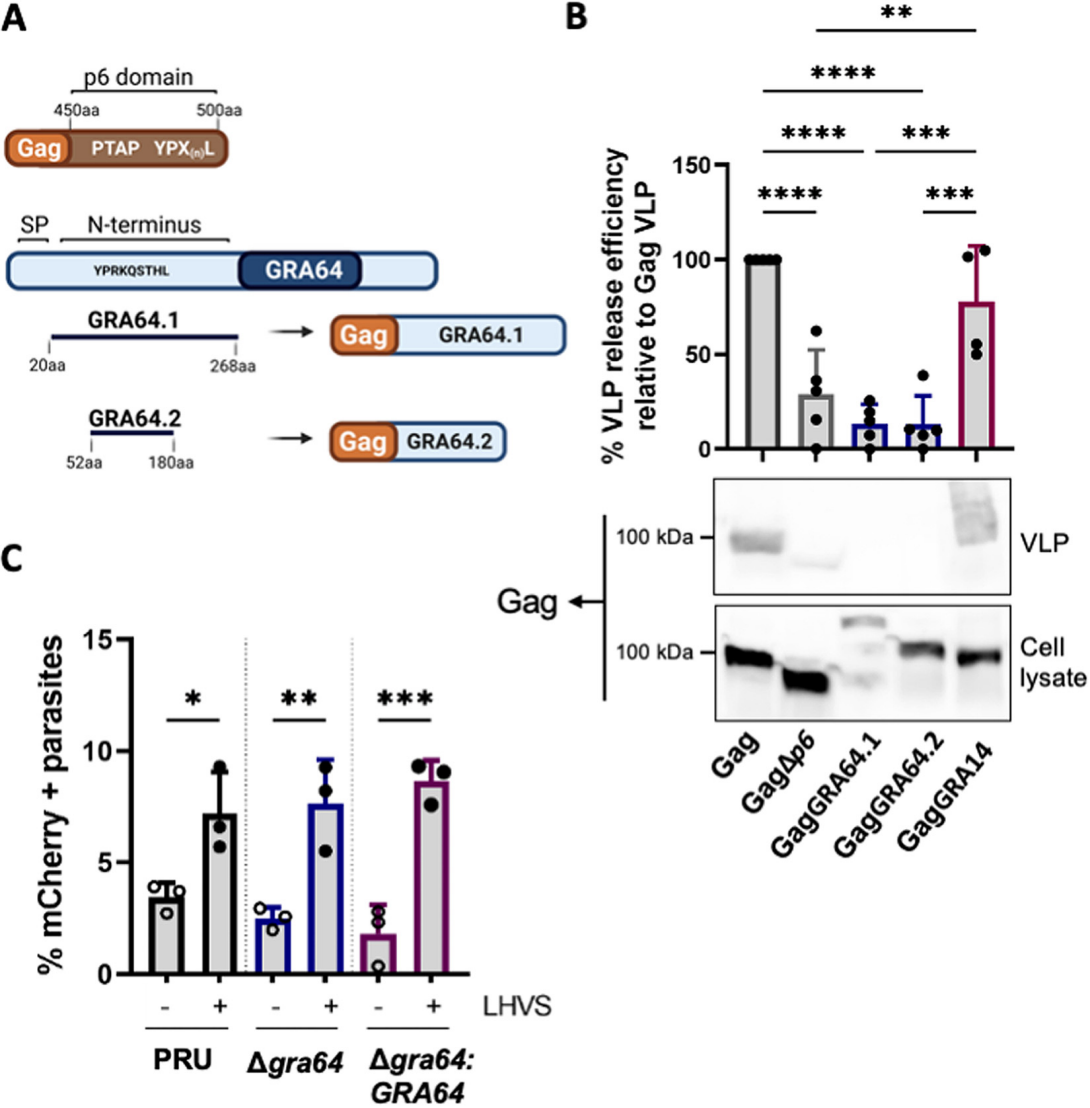
GRA64 does not mediate ESCRT-dependent HIV-1 virus-like particle (VLP) release and is not involved in internalization of host cytosolic proteins. (A) Schematic illustrating the two different GagGRA64 constructs used for experiments in panel B. A putative late-domain motif present in the GRA64 N terminus is indicated. SP, signal peptide. (B) Comparison of percent HIV-1 VLP release efficiency between HIV-1 Gag, GagΔ*p6*, GagGRA64, and GagGRA14 relative to Gag VLP. Data represent means from 4 to 5 biological replicates. Statistical analysis was determined by a one-way ANOVA test followed by Tukey’s multiple-comparison test: **, *P* < 0.01; ***, *P* < 0.001; ****, *P* < 0.0001. (C) Quantification of the uptake of host cytosolic proteins in replicating parasites. Analysis of ingestion by PRUQ, PRUQΔ*gra64*, and PRUQΔ*gra64*::GRA64 parasites treated with dimethyl sulfoxide or LHVS (morpholinurea-leucyl-homophenyl-vinyl sulfone phenyl). At least 200 parasites were analysis per sample. Data represent the means from 3 biological replicates. Statistical analysis was determined by a one-way ANOVA test followed by Tukey’s multiple-comparison test: *, *P* < 0.05; **, *P* < 0.01; ***, *P* < 0.001.

The parasitophorous vacuole transmembrane protein GRA14 can mediate ESCRT-dependent HIV-1 virus-like particle (VLP) budding and internalization of host cytosolic proteins (23). Given the prominent “stalled” intraluminal vesicles within Δ*gra64* tissue cysts, we hypothesized that host ESCRT components are recruited to the parasitophorous vacuole in a GRA64-dependent manner to facilitate vesicle formation. To test this hypothesis, we performed an HIV-1 VLP assay as a functional readout to assess GRA64-dependent ESCRT recruitment for HIV-1 VLP release. Briefly, this assay is performed by transfecting HeLa cells with the constructs necessary to generate HIV-1 virus-like particles (VLPs) and later harvesting VLPs from the supernatant to assess the efficiency of VLP release from HeLa cells. The HIV-1 Gag p6 domain encoding late-domain motifs necessary for ESCRT recruitment was substituted with the GRA64 N terminus capable of interacting with host ESCRT machinery (GagGRA64, Fig. 6A). Two different GagGRA64 constructs were generated: full-length GRA64 protein (GRA64.1) and a truncated GRA64 construct containing only a putative late-domain motif (GRA64.2) (Fig. 6A). Deletion of the HIV-1 Gag p6 domain impaired VLP release as previously observed; however, the expression of either GagGRA64.1 or GagGRA64.2 did not produce VLPs as efficiently as HIV-1 Gag or GagGRA14 (Fig. 6B).

To further assess the role of GRA64 in vesicular trafficking across the PVM, we analyzed the internalization of host cytosolic proteins in GRA64-deficient parasites. Inducible mCherry HeLa cells were infected with type II PRU parasites with (PRUQΔ*gra64*::GRA64) or without GRA64 (PRUQΔ*gra64*). The infected cells were treated with the cathepsin L inhibitor LHVS (morpholinurea-leucyl-homophenyl-vinyl sulfone phenyl) prior to harvesting at 24 h postinfection to allow for accumulation of host-derived mCherry within the parasite’s endolysosomal system, as previously described (13). The percentage of mCherry-containing parasites was not reduced in Δ*gra64* parasites compared to that in complemented parasites, suggesting that GRA64 alone is not required for the uptake of host cytosolic proteins in replicating parasites (Fig. 6C).

## DISCUSSION

*Toxoplasma* serially secretes effector proteins from the micronemes, rhoptries, and dense granule organelles that aid in invasion, immune suppression, vacuole remodeling, and nutrient acquisition (16). Secreted effectors in both tachyzoite and bradyzoite stages traffic to the PVM or cyst membrane/wall and beyond to the host cell cytosol and nucleus (43, 44). Here, we aimed to identify novel effector proteins using an *in silico* screen to identify gene products with similar properties to those of known exported effectors and identified a novel dense granule protein, GRA64. Although GRA64 does not fully translocate across the PVM or cyst membrane into the host cell as a soluble protein, it does appear to reside at the IVN and PVM, with its N terminus exposed to the host cytoplasm. The PVM and cyst membrane are an active parasite/host interface, and we observed that GRA64 co-immunoprecipitates with host ESCRT components and other parasite GRA proteins during tachyzoite and bradyzoite infection. Proximity-based biotinylation with TurboID also demonstrated ESCRT-proximal proteins within living cells during bradyzoite growth conditions. In addition, GRA64-deficient brain cysts exhibited an abnormal cyst wall structure with enlarged vesicular structures. However, unlike GRA14, GRA64 was unable to substitute the HIV-1 Gag ESCRT-interacting domain to mediate VLP release, and GRA64 appears to be dispensable for the internalization of host cytosolic proteins, at least under tachyzoite growth conditions (23). Nonetheless, it is possible that GRA64 recruits the host ESCRT machinery to the parasitophorous vacuole for ESCRT functions at the PVM that remain to be elucidated. This is supported by the fact that the ESCRT accessory protein ALIX is still present at the PVM in GRA14-deficient parasites (23), and by the enlarged vesicular structures at the cyst membrane seen in GRA64-deficient parasites. Thus, we speculate that GRA64 is a membrane-bound GRA protein that helps orchestrate host ESCRT recruitment indirectly under tachyzoite and bradyzoite growth conditions. Co-IP and TurboID experiments confirm interactions between GRA64 and any one of the ESCRT proteins we identified as being enriched by these methods; however, the limitations of studying these interactions at the host PVM or cyst membrane is partially due to the lack of functional assays for these interactions.

GRA64 (*TGME49_202620*) is 330 amino acids in length and possesses a signal peptide (1–19) and a transmembrane domain proximal to the C terminus (amino acids 268 to 291) (40). In a hyperLOPIT subcellular proteomics study, GRA64 was suggested to localize to the dense granule organelles (45), a prediction which we confirmed by colocalization with GRA1 (Fig. 1). GRA64 is expressed in all parasite stages but exhibits high expression in tachyzoites and peak expression in bradyzoites (46). It is notable that *gra64* is conserved in *Hammondia*, *Neospora* (two adjacent genes are present), and *Besnoitia*, which are all cyst-forming coccidians and yet have distinct definitive hosts. Our data suggest that GRA64 behaves as an integral membrane protein following dense granule secretion and is expressed at the PVM, IVN (Fig. 1C and D), and cyst wall (Fig. 2, Fig. 5A). GRA64 is predicted to be intrinsically disordered between the signal peptide and transmembrane domain (Fig. 1B). Disordered regions are dynamic modules that favor protein-protein interactions, and therefore GRA64 could have multiple roles at the IVN, PVM, and cyst membrane. GRA64 has verified phosphorylation sites at residues S59, S80, T81, S216, and S218 and a predicted N-glycosylation site at amino acid 55. It is possible that GRA64 activities are further regulated by protein phosphorylation, N-glycosylation, and/or another post-translational modification.

Toxoplasma gondii resides within a nonfusogenic parasitophorous vacuole derived from the host plasma membrane (47), and this modified membrane, the PVM, is an active site for vesicular trafficking (48, 49). Since the ESCRT machinery is involved in dynamic membrane processes such as membrane remodeling and scission (50), it is likely to be involved in cellular processes at the PVM, e.g., PVM repair or host organelle sequestration into the vacuole at the PVM. Recently, ESCRT accessory host proteins (PDCD6IP/ALIX, PDCD6, and CC2D1A, a regulator of ESCRT-III CHMP4B) were identified in proximity to the PVM using a targeted PVM domain fused to miniTurbo, a variant of biotin proximity-labeling (51). This finding, and those regarding GRA14 (23), leave numerous unanswered and exciting questions about the mechanics of ESCRT molecules at the PVM and cyst membrane and their functional role in the biology of T. gondii. For example, the uptake of host-derived cytosolic proteins occurs at the PVM and is partially dependent on the IVN (13), a membranous network of tubules which are conduits for moving materials such as lipids between the host cell and parasite (52). A recent study demonstrated that several components of host ESCRT-I, ESCRT-III, and ESCRT accessory proteins immunoprecipitated with the vacuolar membrane-localized GRA14 protein in tachyzoite- and bradyzoite-infected cells (23). Furthermore, a direct connection was discovered between ESCRT and GRA14 at the PVM in the mediation of VLP-budding and the ingestion of host cytosolic proteins (23). This discovery demonstrates that, in tachyzoites, host ESCRT proteins interact with a parasite effector protein, GRA14, to regulate membrane vesicular budding and the acquisition of host cytosolic cargo. Our findings on GRA64 interactions with host ESCRT-I, ESCRT-III, and accessory ESCRT proteins demonstrate that GRA14 is likely not the only PVM-bound effector that interacts with the host ESCRT during tachyzoite infection (Fig. 3A). Ongoing and future studies of GRA14 and GRA64 disruption will be essential to determine redundant and/or synergistic roles with host ESCRT in various aspects of nutrient uptake (e.g., recruitment, tethering/binding, ingestion) (53). Furthermore, GRA64 interacts with ESCRT-I, ESCRT-III, and ESCRT accessory proteins during bradyzoite infection of human foreskin fibroblasts (Fig. 3B) and during mouse primary cortical neuron infection (Fig. 3C). Collectively, these observations demonstrate an emerging paradigm in which parasite proteins interact with host ESCRT at the PVM during tachyzoite growth and at the cyst membrane during bradyzoite development. It is probable that there are other parasite effectors that have yet to be characterized and/or discovered which can also interact with the host ESCRT machinery.

The physical nature of GRA64-ESCRT protein interactions is not yet fully known. We have eliminated a functional GRA64-ESCRT interaction via VLP budding assay experiments and show a dispensable role of GRA64 in the acquisition of host proteins from the cytosol during tachyzoite growth conditions (Fig. 6). These results were not entirely surprising as GRA14, unlike GRA64, possesses motifs that closely resemble late-domain motifs, which recruit ESCRT for HIV budding (54–56). Interestingly, a poly-arginine motif in the disordered domain of the HSV-1 protein UL31 has recently been shown to mediate ALIX interaction and ESCRT-III recruitment to the inner nuclear membrane (57). Although a similar motif is present in the GRA64 N terminus from amino acids 230 to 235, it remains to be seen whether this motif universally mediates ALIX interaction and ESCRT-III recruitment across different cellular locales. Given the paucity of previously described ESCRT binding motifs in the GRA64 amino acid sequence, it is also plausible that ESCRT recruitment by GRA64 occurs via ubiquitination (58). ESCRT-I can recognize ubiquitinylated proteins that are targeted for degradation in multivesicular bodies (59). Interestingly, TurboID-GRA64 tachyzoites identified a possible GRA64 interaction with the ubiquitin ligase ITCH (Fig. 3D), which is phosphorylated to promote ubiquitination and subsequent degradation of a substrate (60). ITCH can influence lipid metabolism (61), endocytosis (62), and viral budding and release (63, 64). Using a Bayesian discriminant method to predict ubiquitination positions (65), we observed quite a few predicted ubiquitinated lysine residues in GRA64, with the highest ubiquitination score at K227. This theoretical ubiquitin site faces the host cytosol and is therefore a plausible candidate for ESCRT recruitment. Further studies are required to identify the ubiquitin status of GRA64 and whether this modification is required for ESCRT interactions.

*In vivo* cyst defects due to gene knockouts can be hard to characterize, as knockouts can result in reduced cyst burdens (66, 67) and increased cyst fragility (68), making cyst purification problematic. However, deletion of GRA64 did not significantly reduce cyst burdens (Fig. S4) and therefore, cysts could be further evaluated for ultrastructural changes (Fig. 5). Cysts formed by the Δ*gra64* strain exhibit an intact cyst wall, a normal cyst matrix, and healthy bradyzoites packed with amylopectin. However, the knockout of GRA64 resulted in a more frequent presence of large tubulo-vesicular structures adjacent to the cyst wall, which we interpret as “stalled-scission events,” in which the inefficient or absent recruitment of ESCRT proteins results in the failure of intraluminal vesicle scission into mature tissue cysts. Results from the robust quantification of tubulo-vesicular structures in cysts of various ME49Q strains corroborate a tendency for these enlarged structures to arise more frequently in the ME49QΔ*gra64* strain (Fig. 5C and D, Fig. S6). The directionality of these enlarged tubulo-vesicular structures (i.e., whether they are derived from or being targeted to the cyst membrane) and the possible consequences of stalled-scission events are unknown and demand further investigation.

It is not surprising that host ESCRT molecules are intimately involved in T. gondii life stages, as the parasite recruits organelles and acquires nutrients from the host such as lysosomes (18), lipids (69), and cholesterol (70). The recent discovery of ESCRT interactions at the PVM and cyst membrane by independent labs corroborates the connection and highlights the complexity of this process. Our findings hint at a possible role for host ESCRT recruitment at mature *in vivo* cyst membranes. This is only the beginning with respect to understanding the relevance of ESCRT recruitment at tachyzoite and bradyzoite vacuolar membranes.

## MATERIALS AND METHODS

### Cell culture.

All parasite strains were continuously passaged in human foreskin fibroblasts (HFF:ATCC:CRL-1634; Hs27) in a 37°C, 5% CO_2_ incubator using Dulbecco’s modified Eagle medium (DMEM, Gibco) supplemented with 10% fetal calf serum, 1% l-glutamine, and 1% penicillin and streptomycin. Cultures were regularly inspected and tested negative for mycoplasma contamination. Bradyzoite induction was performed at the time of invasion by replacing growth media with bradyzoite induction media (50 mM HEPES [pH 8.2], DMEM supplemented with 1% fetal bovine serum [FBS], penicillin, and streptomycin) prior to infection of human foreskin fibroblasts with egressed tachyzoites. Bradyzoite-induced cultures were maintained in a 37°C incubator without CO_2_, with induction medium replaced every 2 days for all experiments.

Mouse primary cortical neurons were harvested from E14 mouse embryos obtained from pregnant C57BL/6 mice ordered from Charles River Laboratories or Jackson Laboratories. Dissections of E14 cortical neurons were performed as previously described (71). Following dissection, 1 × 10^6^ to 1 × 10^7^ cortical neurons were plated onto poly-l-lysine-coated, 15-cm diameter culture dishes and later cultured in Neurobasal Medium (Thermo Fisher Scientific, San Jose, CA) supplemented with GlutaMAX Supplement (Thermo Fisher) and B-27 Supplement (Gibco, Waltham, MA). After 4 days *in vitro* (DIV), cytarabine (ara-C) was added to each culture at a final concentration of 0.2 μM to minimize contamination from dividing, non-neuronal cells. Cultures were maintained for up to 16 days by replacing half of the conditioned medium with fresh supplemented Neurobasal Medium every 7 days.

### Cloning and parasite transfections.

For a full list of primers used for cloning and genetic manipulations, please refer to Table 1. Briefly, for all Cas9-mediated genetic manipulations, single guide RNAs (sgRNA) targeting the N terminus of GRA64 were cloned into the p-HXGPRT-Cas9-GFP plasmid backbone using KLD (Kinase, Ligase, and Dpnl) reactions (New England Biolabs, Ipswich, MA), as previously described (72). Next, 100-bp donor oligonucleotides were designed and synthesized (Thermo Fisher) with homologous arms targeting the region of interest and encoding either an epitope tag, stop codons (inserted in place of the start codon for knockout transfections), or a start codon (inserted in place of the ectopic stop codon in the knockout strain for complement transfections), all in-frame with the region of interest. Donor sequences for homology-mediated recombination with TurboID were generated by PCR using 3×HA-TurboID-NLS_pCDNA3 (kind gift from Alice Ting, Addgene plasmid no. 107171) as the plasmid template, with primers containing overhangs with 40-bp homology to the GRA64 region of interest.

**TABLE 1:** List of primers used in this study for CRISPR/Cas9 tagging and cloning of GRA64^*a*^

Primer	Sequence (5′–3′)	Purpose
gRNA_R	AACTTGACATCCCCATTTAC	KLD sgRNA cloning
KLD_202620_NTerm_gRNA_FWD	GGCTCTACCAGGAGGCGCGTGTTTTAGAGCTAGAAATAGC	KLD sgRNA cloning
KLD_GRA64_KO_gRNA_FWD	GAATCCTCGACTCCACCGGGGTTTTAGAGCTAGAAATAGC	KLD sgRNA cloning
KLD_COMP_gRNA_FWD	TGACTAACTAGCTAACTAGGGTTTTAGAGCTAGAAATAGC	KLD sgRNA cloning
GRA64NTerm_3×HA_FWD	CGTCCGGTTTACGCGCTCCTGGCTCTACCAGGAGGAGCCTTAGCGGGCGGATACCCGTACGACGTCCCGGA	Endogenous tagging
GRA64NTerm_3×HA_RVS	TTCCTGCTGGTTGCCCAAGAACAAAGAAATCGTAGTTTGCGGCGGAGGCATAATCTGGAACATCGT	Endogenous tagging
GRA64NTerm_HA_FWD	CGTCCGGTTTACGCGCTCCTGGCTCTACCAGGAGGAGCCTTAGCGGGCGGATACCCGTATGATGTTCCGGA	Endogenous TurboID tagging
GRA64NTerm_TurboID_RVS	TTCCTGCTGGTTGCCCAAGAACAAAGAAATCGTAGTTTGCGGCGGACTTTTCGGCAGACCGCAGAC	Endogenous TurboID tagging
GRA64_KO_Donor_FWD	CCTGCCCAATAACCCGCCCGGTGGAGTCGAAGCTGCGATTGACTAACTAGCTAACTAGGGGGAAGTTATTCCGTCCGGTTTACGCGCTCCTGGCTCTACC	KO donor oligonucleotide
GRA64_KO_Donor_RVS	GGTAGAGCCAGGAGCGCGTAAACCGGACGGAATAACTTCCCCCTAGTTAGCTAGTTAGTCAATCGCAGCTTCGACTCCACCGGGCGGGTTATTGGGCAGG	KO donor oligonucleotide
GRA64_Comp_Donor_FWD	TGTGATCTTCCTGCCCAATAACCCGCCCGGTGGAGTCGAGGATTCGATATG AAATTATTTCGTCCGGTTTACGCGCTCCTGGCTCTACCAGGAGGCGCGT	COMP donor oligonucleotide
GRA64_Comp_Donor_RVS	ACGCGCCTCCTGGTAGAGCCAGGAGCGCGTAAACCGGACGAAATAATTTCATATCGAATCCTCGACTCCACCGGGCGGGTTATTGGGCAGGAAGATCACA	COMP donor oligonucleotide
GRA64_5UTR_FWD	ATTGGCGTGGCGAGTCCCTT	Confirm gDNA editing
GRA64_NTerm_RVS	ATCCTTGTTCGAGGTCTTTT	Confirm gDNA editing

a KLD, Kinase, Ligase, and Dpnl; sgRNA, single guide RNA.

For all transfections, 5 × 10^6^ to 1 × 10^7^ PruΔ*ku80*Δ*hxgprt* or ME49Δ*ku80*Δ*hxgprt* tachyzoites were electroporated in CytoMix (73) after harvesting egressed parasites from human foreskin fibroblast monolayers and filtering through 5-μm filters. For each transfection, 7.5 μg of uncut Cas9 plasmid and 1.5 μg of PCR-amplified donor sequence or 280 pmol unannealed 100-bp donor oligonucleotides was used. Selection of transfected parasites was performed with medium containing 25 μg/mL mycophenolic acid and 50 μg/mL xanthine 24 h posttransfection for 6 days before removing selection medium and subcloning by limiting dilution after sufficient parasite egress had been observed.

### Immunofluorescence assays.

Human foreskin fibroblast monolayers were grown to confluence on glass coverslips and infected with egressed tachyzoites at a multiplicity of infection (MOI) of 1 for most immunofluorescence assays, allowing growth to proceed under tachyzoite or bradyzoite growth conditions (using the medium formulation described above). All coverslips were fixed with 4% paraformaldehyde (PFA) for 20 min at room temperature, permeabilized in a 0.2% Triton X-100, 0.1% glycine solution for 20 min at room temperature, rinsed with PBS, and blocked in 1% BSA for either 1 h at room temperature or at 4°C overnight. For selective permeabilization experiments, coverslips were fixed with 4% PFA for 20 min, allowed to cool in PBS at 4 C° for 15 min, incubated in 0.001% digitonin in PBS for 5 min at 4 C°, detergent-rinsed with PBS, and blocked in 1% BSA for 1 h at room temperature or overnight. After blocking, coverslips were labeled with antibodies as follows: HA-tagged proteins were detected with rat anti-HA 3F10 (Sigma-Aldrich, 1:200 to 1:500), parasite membrane with mouse anti-SAG1 (Thermo Fisher, 1:250), parasitophorous vacuole with in-house mouse anti-MAG1 (MAb bB6; 1:500), dense granules with in-house mouse anti-GRA1 (MAb 92.10B; 1:1000), and biotin with anti-biotin (Abcam, ab53494, 1:1,000). Appropriate secondary antibodies conjugated to Alexa Fluorophores 488, 555, 594, and 633 targeting a given primary antibody species, or streptavidin conjugated to Alexa Fluorophore 488, were used at a dilution of 1:1,000 (Thermo Fisher). A 4′,6-diamidino-2-phenylindolecounterstain was used to label parasite and host cell nuclei (1:2,000). Coverslips were mounted in ProLong Gold Anti-Fade Reagent (Thermo Fisher) and imaged using either a Leica SP8 confocal microscope or a Nikon Eclipse widefield fluorescence microscope (Diaphot-300).

### Membrane fractionation.

Human fibroblast monolayers were infected with parasites and cultured for 1 day under tachyzoite growth conditions. The infected cells were washed and scraped with ice-cold PBS (containing inhibitor cocktail with EDTA, 5 mM NaF, and 2 mM activated Na_3_VO_4_). Infected cultures were lysed by passage through a 27-gauge needle, and the bulk of intact parasites was removed by low-speed centrifugation at 2,500 × *g* for 10 min at 4°C. The resulting low-speed pellet was resuspended in radioimmunopreciptation assay (RIPA) buffer for immunoblot assessment, while the low-speed supernatant was separated into soluble and membrane-enriched fractions by high-speed centrifugation at 100,000 × *g* for 1.5 h. The resulting high-speed supernatant (HSS) containing soluble parasitophorous vacuole components was saved, while the resulting high-speed pellets (HSP) were treated by resuspension in various buffers and incubated on ice for 45 min. Resuspended fractions were centrifuged again at 100,000 × *g* for 1.5 h to separate solubilized proteins in the high-speed supernatant from remaining membrane-bound proteins in the high-speed pellet. All high-speed supernatant fractions were concentrated by either acetone precipitation overnight or deoxycholate-trichloroacetic acid precipitation prior to immunoblotting.

### Immunoblotting.

Protein lysates were prepared in radioimmunopreciptation assay buffer from infected fibroblast cultures as specified for each experiment. Laemmli sample buffer was added to all samples and boiled for 5 min before loading onto an SDS-PAGE 4 to 20% or 4 to 15% precast gradient gel (TGX). Transfer to polyvinylidene difluoride membranes (Millipore) was performed in Towbin buffer (20% methanol, Tris/glycine) for 2 h at 100 V, and blocking in 5% BSA/TBST (TBST, Tris-buffered saline with Tween 20) was performed overnight in 4°C. Membranes were labeled in 5% BSA/TBST with either anti-HA peroxidase-conjugated antibodies (Sigma-Aldrich, 1:200), mouse GRA5 antibody (mAbTG17.113 BioVision cat no. A1299, 1:8,000, kind gift from Vernon Carruthers) and anti-mouse horseradish peroxidase (HRP) antibodies (Thermo Fisher, 1:10,000), rabbit GRA1 antibody (1:10,000, kind gift from Vernon Carruthers), rabbit GRA14 antibody (1:10,000, kind gift from Vernon Carruthers), rabbit TgALD1 antibody (1:200, kind gift from Kentaro Kato) or rabbit SAG1 antibody (1:500, kind gift from Vernon Carruthers) and anti-rabbit HRP antibodies (Thermo Fisher, 1:10,000). West Pico Plus Chemiluminescent Substrate (Thermo Fisher) was used to develop signals from HRP secondary antibodies. Alternatively, LiCor anti-rabbit 680 and LiCor anti-mouse 800 secondary antibodies were used. Antibodies against TSG101 (Invitrogen Clone 4A10, MA1-23296) and PDCD6 (Proteintech, 12303-1-AP) were also used. Images of labeled blots were collected with a Li-COR instrument (Odyssey Imaging System) or a Bio-Rad Chemidoc Imaging System.

### Immunoelectron and transmission electron microscopy.

For immunoelectron microscopy of 3×HA-GRA64 PruQ-tagged tachyzoites, samples were prepared from infected human fibroblast monolayers grown under tachyzoite growth conditions for 24 h. Cultures were fixed in 4% paraformaldehyde and immunolabeled with anti-HA antibody conjugated to both Alexa Fluorophore 488 and a 10-nm gold particle using the protocol described above, including the use of 0.2% Triton X-100. After imaging Alexa Fluorophore 488-labeled GRA64, cells were fixed with 2.5% glutaraldehyde and 2.0% paraformaldehyde in 0.1 M sodium cacodylate buffer, rinsed with 0.1 M sodium cacodylate buffer, postfixed with 1% osmium tetroxide, *en bloc* stained with 2% uranyl acetate, dehydrated in a graded series of ethanol, and infiltrated with LX112 epoxy resin (LADD Research Industries, Burlington, VT). Samples were polymerized at 60°C for 60 h and blocks were popped-off the coverslip. Regions of interest (ROI) were cut out and remounted on a flat BEEM capsule for sectioning. Trimming to the specific ROI was done using Trimtool 45° (Diatome) blocks, then serial thin-sectioned (70 nm) *en face* on a Leica UC7 using a Diatome Ultra 35° knife. Sections were picked up on Formvar-coated slot grids, stained with uranyl acetate and lead citrate, and photographed using Kodak 4489 film on a JEOL 1200 EX transmission electron microscope (TEM).

For transmission electron microscopy of *in vivo*-derived tissue cysts, cysts were harvested from homogenized infected mouse brains (4 weeks postinfection) using a Wheaton Potter-Elvehjem Tissue Grinder. Cysts were enriched with 45% Percoll and centrifugation at 26,600 × *g* for 20 min at 4°C. The cyst enriched fraction was harvested from the Percoll solution and diluted in PBS prior to a final spin at 130 × *g* for 10 min at 4°C. Pellets containing cysts were resuspended in either 2.5% glutaraldehyde and 2% paraformaldehyde in 0.1 M sodium cacodylate buffer for morphological analysis or in 4% paraformaldehyde and 0.1% glutaraldehyde in 0.1 M sodium cacodylate buffer for immunoelectron microscopy. Samples for morphological analysis were prepared as described above prior to imaging with a JEOL 1400 EX transmission electron microscope, whereas samples prepared for immunoelectron microscopy were dehydrated with a graded ethanol series, embedded in Lowicryl HM-20 Monostep resin, and polymerized by UV light prior to labeling with anti-HA antibody (Roche, clone 3F10, 1:40 dilution of a 200 μg/mL stock) and 10-nm gold bead-conjugated goat anti-rat (Electron Microscopy Sciences, 1:100 dilution), or with goat anti-rat only as a negative control. Electron microscopy images of *in vivo*-derived tissue cysts were viewed on a JEOL 1400 Plus using Digital Micrograph software from Gatan.

For tomograms of *in vivo* derived tissue cysts, 250-nm thick epoxy sections were picked up onto slot grids and post-stained with uranyl acetate and lead citrate. Gold fiducial markers (10 nm) were added to the sample prior to imaging to aid in downstream alignment. Tilt series (single axis) were collected on the JEOL 1400 Plus from −60 to +60 using Serial EM software (74). IMOD software was used for the alignment and reconstruction of each tomogram, as well as for the tracing of structures (e.g., vesicles, cyst wall, and parasite membrane) observed in the tomogram to create a model (75).

### Quantitative tubulo-vesicular structure analysis.

For quantitative TEM image analysis, all tubulo-vesicular structures within the cyst wall (defined as the luminal electron dense region adjacent to the cyst membrane) were manually traced in ImageJ from blinded TEM thin-section images acquired from *in vivo* tissue cysts at 5,000× magnification. Tracings were made only from clearly identifiable circumscribed structures or tubular invaginations of the cyst membrane using the polygon tool in ImageJ, from which Area, Feret Diameter, and Circularity measurements were obtained. In the case of cyst membrane invaginations, a line parallel to the cyst membrane at the site of invagination was drawn to obtain a region of interest. Large vesicular structures containing fibrillary or granular material were excluded from analysis, as these structures were felt to differ in nature from the cyst membrane invaginations observed by electron tomography. At least five separate 5,000× fields of view were obtained from each cyst. Cysts from each strain were analyzed from at least two separate mouse infection experiments. One-way analysis of variance (ANOVA) or Kruskal-Wallis tests were used for statistical analysis for normal and non-normal distributions, respectively, in GraphPad Prism 9.

### Plaque assays.

Parasites were harvested from host cells with a 27G needle and filtered through a 5-μm filter to remove host cell debris. Parasite numbers were quantified with a hemocytometer, and 100 parasites from each strain were added in triplicate to wells containing confluent human foreskin fibroblasts in 6-well dishes. Parasites were grown for 14 days before fixing and staining with a 20% methanol-0.5% crystal violet solution. Plaque size was quantified from blinded images using ImageJ and the line tool to separate neighboring plaques. Kruskal-Wallis tests with Dunn’s multiple comparisons were performed to test for significant differences in average plaque size with PRISM 8. Plaque assays were repeated three times with PruQ GRA64 parasite strains, using different batches of parasites and host cells for each independent experiment.

### Co-immunoprecipitations.

For GRA64 Co-IPs from tachyzoite-infected cultures, two independent experiments were performed in which 15-cm diameter cell culture dishes containing confluent human foreskin fibroblast monolayers were infected at an MOI of 3 with either parasite expressing a 3×HA tag at the endogenous locus of GRA64 (N terminus), using an equivalent amount of PruQ non-HA-tagged parasites each replicate as a control in parallel. Dishes were washed with ice-cold PBS at 24 h postinfection and lifted off each dish with a cell scraper in 1 mL ice-cold lysis buffer [50 mM Tris (pH 7.4), 200 mM NaCl, 1% Triton X-100, and 0.5% CHAPS (3-[(3-cholamidopropyl)-dimethylammonio]-1-propanesulfonate)] supplemented with cOmplete EDTA-free protease inhibitor (Sigma-Aldrich) and phosphatase inhibitors (5 mM NaF, 2 mM activated Na_3_VO_4_). Scraped cultures were passed through a 27G needle five times and sonicated for 30 s total (20% amplitude, 1-s pulses). Sonicated samples were incubated on ice for 30 min, the supernatant cleared by centrifugation (1,000 × *g*, 10 min), and then samples were incubated overnight in a 4°C rotator with 0.25 mg anti-HA magnetic beads (100 μL slurry, Thermo Fisher). Following overnight incubation, beads were separated on a magnetic stand and washed twice in lysis buffer and four times in wash buffer (50 mM Tris pH 7.4, 300 mM NaCl, 0.1% Triton X-100) prior to elution in Laemmli buffer with 50 mM DTT, boiling beads for 5 min prior to magnetic separation and collection of eluates. Eluates were loaded, washed, and digested into peptides with 1 μg of trypsin on S-TRAP micro columns (ProtiFi, Farmingdale, NY) per manufacturer guidelines. S-TRAP peptide eluates were concentrated with a speed vac, desalted in HLB resin (Waters), and concentrated in a speed vac once more prior to LC-MS/MS acquisition.

For GRA64 Co-IPs from bradyzoite and neuron infection conditions, two separate experiments were performed identically to that described above for tachyzoite Co-IPs, except that bradyzoite infections in human fibroblast monolayers were performed at an MOI of 1 in bradyzoite differentiation medium and cultures were maintained for 4 days of infection prior to protein harvesting. For neuron infections, an MOI of 3 and an infection period of 2 days were used prior to protein harvesting, infecting neurons after 14 DIV.

For the reciprocal Co-IP using CHMP4B antibody (Thermo Fisher, rabbit polyclonal, cat no. PA5-64271), 7.5 μg of antibody was conjugated to Dynabeads per the manufacturer’s guidelines. Two 15-cm dishes were infected with 3×HA-GRA64-tagged PruQ parasites at an MOI of 3, with infection proceeding under tachyzoite growth conditions for 24 h. Protein was harvested and Co-IPs performed as described above with either CHMP4B-conjugated Dynabeads or unconjugated Dynabeads (with an equivalent weight to that used for CHMP4B antibody conjugation). Eluates were collected and analyzed by immunoblotting, as described above.

### Proximity-based biotinylation protein preparation.

For GRA64-TurboID proximity-based biotinylation experiments, untagged PruQ parasites or parasites expressing TurboID-tagged GRA64 were used to infect either primary cortical neurons or confluent human fibroblast monolayers in two 15-cm dishes at an MOI of 1 (under bradyzoite growth conditions for fibroblast infections, as described above) for 3 days per biological replicate. Exogenous biotin was supplemented to the medium at a final concentration of 150 μM during the 3-day culture period. Following infection with biotin supplementation, dishes were rinsed, scraped, and pelleted in PBS (500 × *g*, 10 min), after which pellets were solubilized in RIPA buffer supplemented with protease inhibitor cocktail (Roche cOmplete tablets). After 30 min of RIPA buffer incubation on ice, insoluble material was cleared from supernatant by centrifugation (16,000 × *g* for 15 min), and supernatant was incubated with streptavidin agarose resin (Thermo Fisher) overnight at 4 C° on a rotator. Following incubation, streptavidin resin and bound biotinylated proteins were washed in RIPA urea buffer (50 mM Tris-HCl [pH 7.5], 8 M urea, 150 mM NaCl) and subsequently reduced and alkylated with Tris(2-carboxyethyl)phosphine hydrochloride (TCEP)-HCl and iodoacetamide, respectively. On-bead digestion was performed with trypsin and Lys-C proteases, and peptides were harvested from streptavidin resin. Peptides were desalted using C18 tips (Thermo Fisher) prior to liquid chromatography-tandem mass spectrometry acquisition.

### LC-MS/MS acquisition and analysis.

For peptide samples from all Co-IP and GRA64-TurboID experiments, samples were resuspended in 10 μL of water + 0.1% trifluoroacetic acid and loaded onto a Dionex RSLC Ultimate 300 (Thermo Fisher Scientific, San Jose, CA), coupled online with an Orbitrap Fusion Lumos (Thermo Fisher Scientific). The mass spectrometer was set to acquire spectra in a data-dependent acquisition mode. Briefly, the full MS scan was set to 300 to 1,200 *m/z* in the orbitrap with a resolution of 120,000 (at 200 *m/z*) and an AGC target of 5 × 10e^5^. MS/MS was performed in the ion trap using the top speed mode (2 secs), an AGC target of 10e^4^, and a higher energy collision dissociation energy of 30. Raw files were searched using Proteome Discoverer software (v2.4, Thermo Fisher Scientific) using SEQUEST as the search engine. We used the Swiss-Prot human or mouse databases (updated January 2020) and the *Toxoplasma* database (release 44, ME49 proteome obtained from ToxoDB). The search for total proteome included variable modifications of methionine oxidation and N-terminal acetylation, and fixed modification of carbamidomethyl cysteine. Trypsin was specified as the digestive enzyme. Mass tolerance was set to 10 pm for precursor ions and 0.2 Da for product ions. Peptide and protein false discovery rates were set to 1%.

For quantitative analysis, peptide intensity values were log_2_-transformed and normalized by the average value of each sample, and missing values were imputed using a normal distribution 2 standard deviations lower than the mean. Individual peptide fold changes (Tag versus Control) for a given protein were calculated and averaged to obtain protein fold enrichment. *P* values were then obtained from *t*-distributions, and *t*-values calculated for each protein with at least two detected peptides by treating protein fold enrichment as the sample mean and using log-transformed peptide intensity values to calculate the standard deviation, sample size, and degrees of freedom. Data distribution was assumed to be normal, but this was not formally tested. Fold change and *P* value significance cutoffs for both Co-IP and TurboID experiments were arbitrarily selected.

LC-MS/MS data from both Co-IP and TurboID experiments have been deposited onto the public repository Chorus under Project ID 1735.

### Mouse experiments.

Eight-week-old female C57BL/6 mice (The Jackson Laboratory, Bar Harbor, ME) were infected intraperitoneally with 2,000 tachyzoites of each strain for all acute virulence/survival curve experiments. Mortality was observed daily over 30 days. For cyst burden analysis, brains were collected from C57BL/6 mice at 28 to 30 days postinfection and from CBA/J mice (The Jackson Laboratory, Bar Harbor, ME) at 28 to 30 days postinfection. One brain hemisphere or a whole brain from an infected mouse was homogenized using a Wheaton Potter-Elvehjem Tissue Grinder with a 100- to 150-μm clearance (Thermo Fisher) in PBS, and an aliquot of the homogenate was viewed under an epifluorescence microscope (Nikon) to count green fluorescent protein (GFP)-positive cysts. Kruskal-Wallis tests and Dunn’s multiple-comparison test were performed to test for significance between groups with non-normal distribution using Prism 8. For groups with normal data distribution, a one-way ANOVA test was used to determine statistical significance. A log-rank test was performed to test for statistical significance in Kaplan-Meier survival curves in Prism 8.

### HIV-1 virus-like particle assay.

To generate GagGRA64_Venus, pGag_Venus was linearized using SwaI and SmaI to delete the Gag p6 domain (amino acids 450 to 500), which functions in the interaction with host ESCRT machinery through the PTAP and YPXL motifs. Two GRA64 constructs were designed for the substitution of the Gag p6 domain. The first fragment encompasses the entire GRA64 N terminus from amino acids 20 to 268 (GRA64.1), and the second fragment was amplified from amino acids 52 to 108 (GRA64.2), comparable in size to the fragment designed to generate the GagGRA14 construct encoding putative late-domain motifs (23). Fragments were introduced into the linearized Gag_Venus plasmid vector using Gibson Assembly. All plasmids were confirmed by Sanger sequencing.

HIV-1 Gag virus-like particles were collected by ultracentrifugation and analyzed by immunoblotting as previously described (23, 76). Lipofectamine 2000 was used to transfect HeLa cells with pRev, pVphu, and pGag_Venus constructs. At 18 h posttransfection, the supernatants containing the released VLPs were collected. The samples were filtered and ultracentrifuged at 35,000 rpm for 45 min at 4°C to collect the VLP pellets, which were further lysed with 0.5% Triton X-lysis buffer. The cell lysates were prepared by lysing the transfected monolayer with the same lysis buffer. The lysates (obtained by loading 100% VLP fraction and 6% cellular fraction on SDS-PAGE gel) were analyzed using immunoblotting by probing with human anti-Gag. Band intensity was quantified using Image J. Total Gag corresponds to the sum of cell- and VLP-associated Gag. The VLP release efficiency corresponds to the fraction of Gag that was released as VLP relative to the total Gag. The percentage of VLP release is set to 100% and normalized relative to Gag, which had a % release efficiency of 5.57 ± 3.6.

### Parasite ingestion assay.

Inducible mCherry HeLa cells (previously described [23]) were seeded in a 6-well plate and induced for 4 days for cytosolic mCherry expression by adding 2 μg/mL doxycycline. The cells were then infected with 1 × 10^6^ parasites and treated with 5 μM LHVS (morpholinurea-leucyl-homophenyl-vinyl sulfone phenyl) at 4 h postinfection to inhibit the degradation of ingested material. Parasites were harvested at 24 h postinfection as previously described (13).

### Ethics statement.

All mouse experiments were conducted according to guidelines from the United States Public Health Service Policy on Humane Care and Use of Laboratory Animals. Animals were maintained in an AAALAC-approved facility, and all protocols were approved by the Institutional Care Committee of the Albert Einstein College of Medicine, Bronx, NY (Animal Protocol 00001451; Animal Welfare Assurance no. A3312-01).

10.1128/mbio.01442-22.9 DATA SET S1 LC-MS/MS data from Co-IP experiments. Data are shown in 11 different tabs. The “Summary” tab for tachyzoite, bradyzoite, and neuron lists the calculated protein fold change (GRA64-3xHA/Control) and log_2_ *P* values for all proteins detected in each replicate. The “Replicate 1/2 Analysis” tab for tachyzoite, bradyzoite, and neuron shows data transformation steps and equations used to determine average protein fold change and log_2_ *P* values. The “Replicate 1/2 Raw Data” tab provides information on search engine identification quality parameters. This dataset has been deposited into the mass spectrometry open access repository Chorus under Project ID 1735. Download Data Set S1, XLSX file, 11.7 MB. Copyright © 2022 Mayoral et al. 2022 Mayoral et al. https://creativecommons.org/licenses/by/4.0/ This content is distributed under the terms of the Creative Commons Attribution 4.0 International license.

10.1128/mbio.01442-22.10 DATA SET S2 LC-MS/MS data from TurboID experiments. Data are shown in seven different tabs. The “Results Summary” tab for human foreskin fibroblast and neuron lists the calculated protein fold change (TurboID-GRA64/Control) and log_2_ *P* values for all proteins detected in each replicate. The “Replicate 1/2 Analysis” tab for human foreskin fibroblast and neuron demonstrates data transformation steps and equations used to determine average protein fold change and log_2_ *P* values. The “Raw Data” tab provides information on search engine identification quality parameters for replicates 1 and 2. This dataset has been deposited into the mass spectrometry open access repository Chorus under Project ID 1735. Download Data Set S2, XLSX file, 13.2 MB. Copyright © 2022 Mayoral et al. 2022 Mayoral et al. https://creativecommons.org/licenses/by/4.0/ This content is distributed under the terms of the Creative Commons Attribution 4.0 International license.
