# Supplementary figures and images for: Dense Granule Protein GRA64 Interacts with Host Cell ESCRT Proteins during Toxoplasma gondii Infection
Source: mBio. 2022 Jun 22;13(4):e01442-22. doi: 10.1128/mbio.01442-22 (PMC9426488; doi:10.1128/mbio.01442-22)

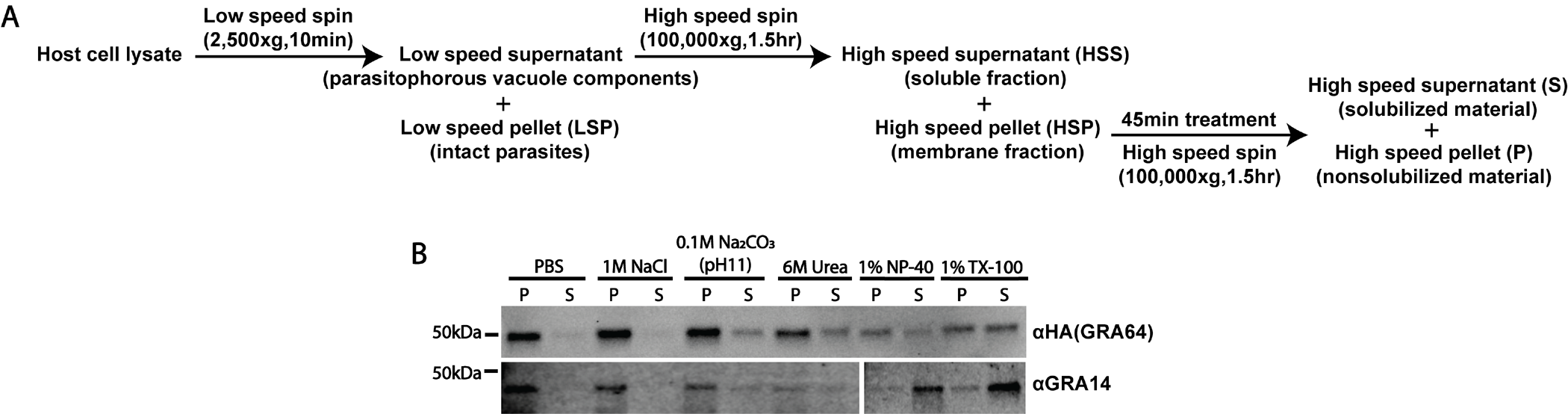

Supplement: FIG S1 [file mbio.01442-22-s0003.tif]

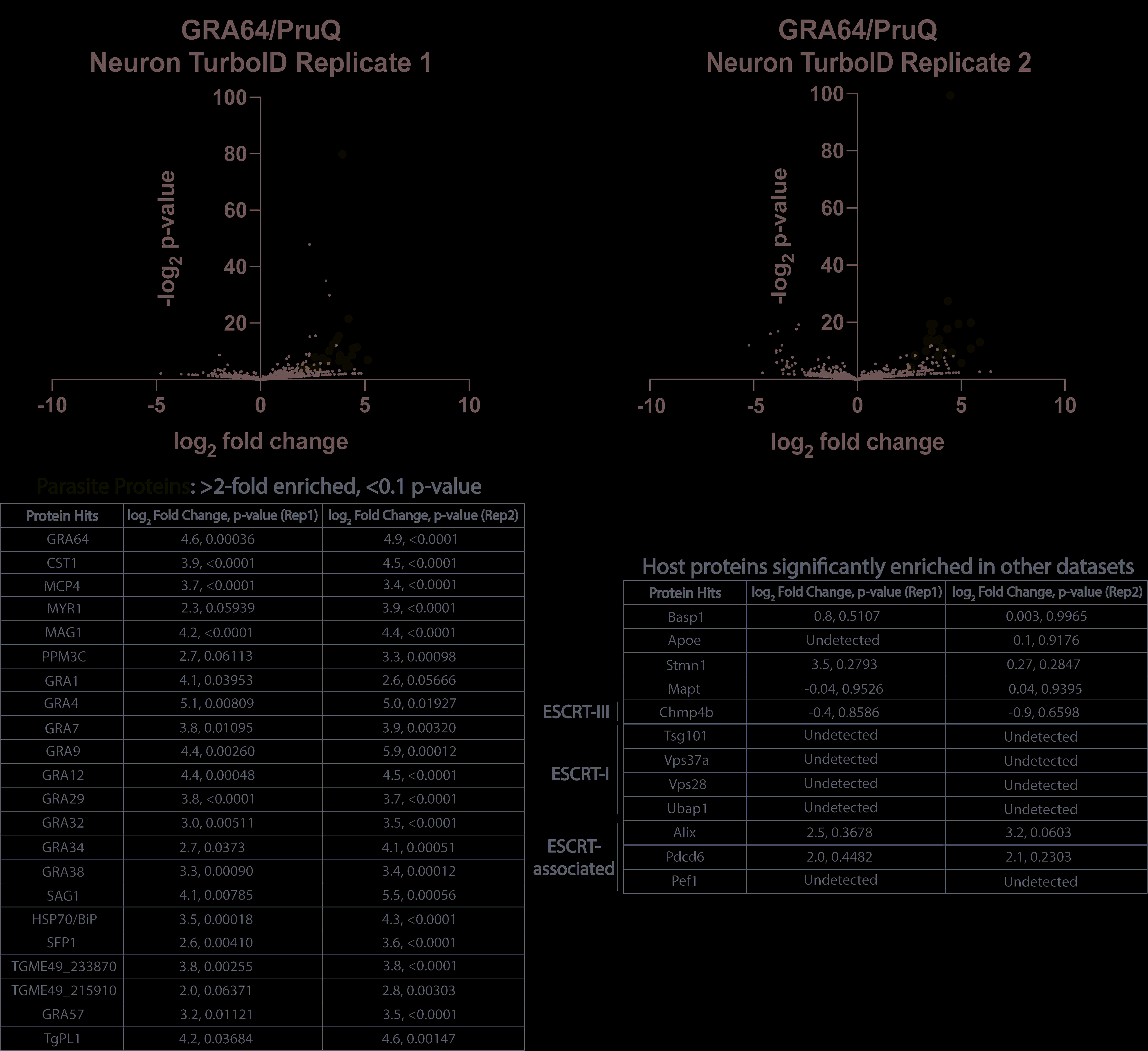

Supplement: FIG S2 [file mbio.01442-22-s0004.tif]

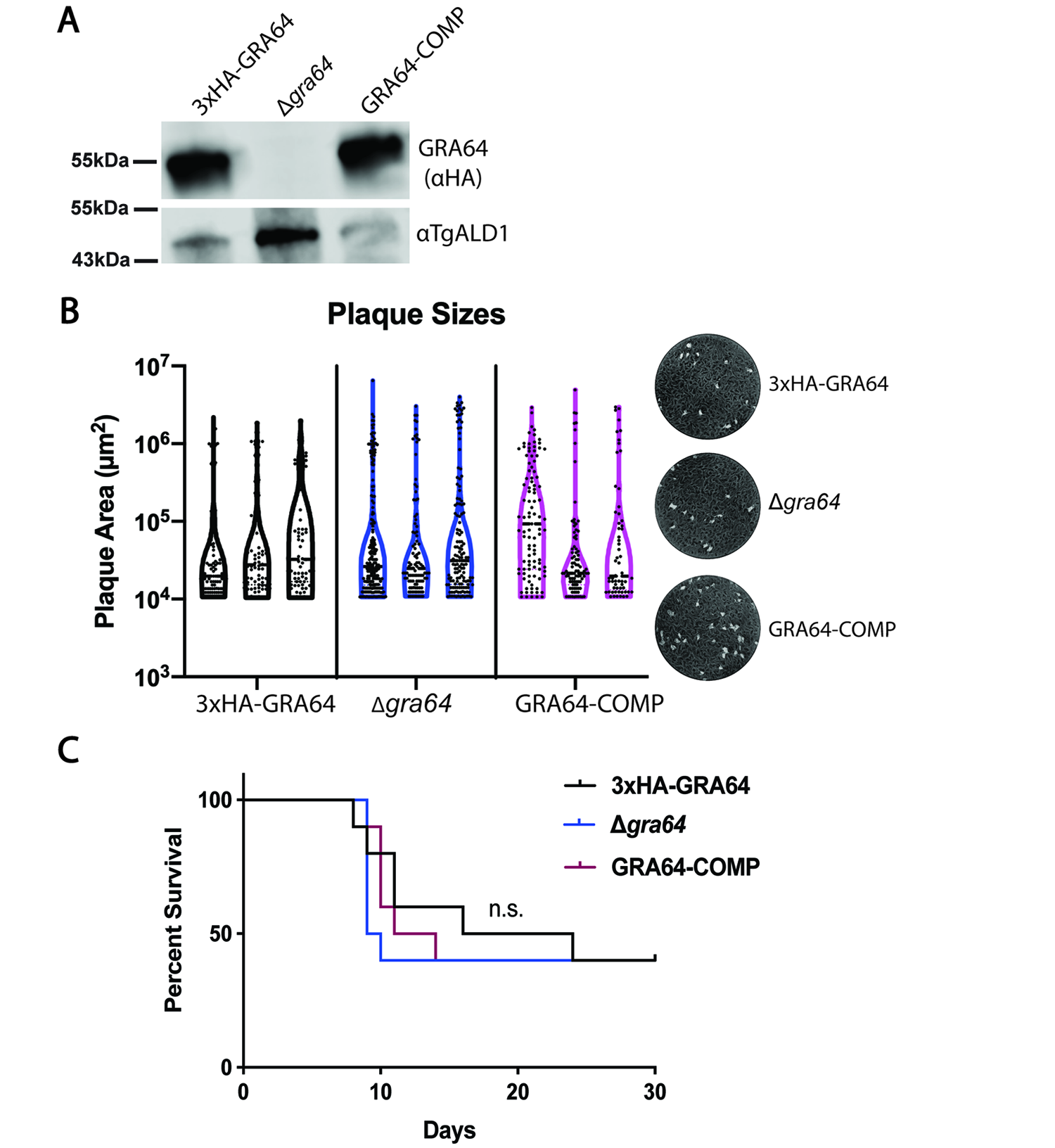

Supplement: FIG S3 [file mbio.01442-22-s0005.tif]

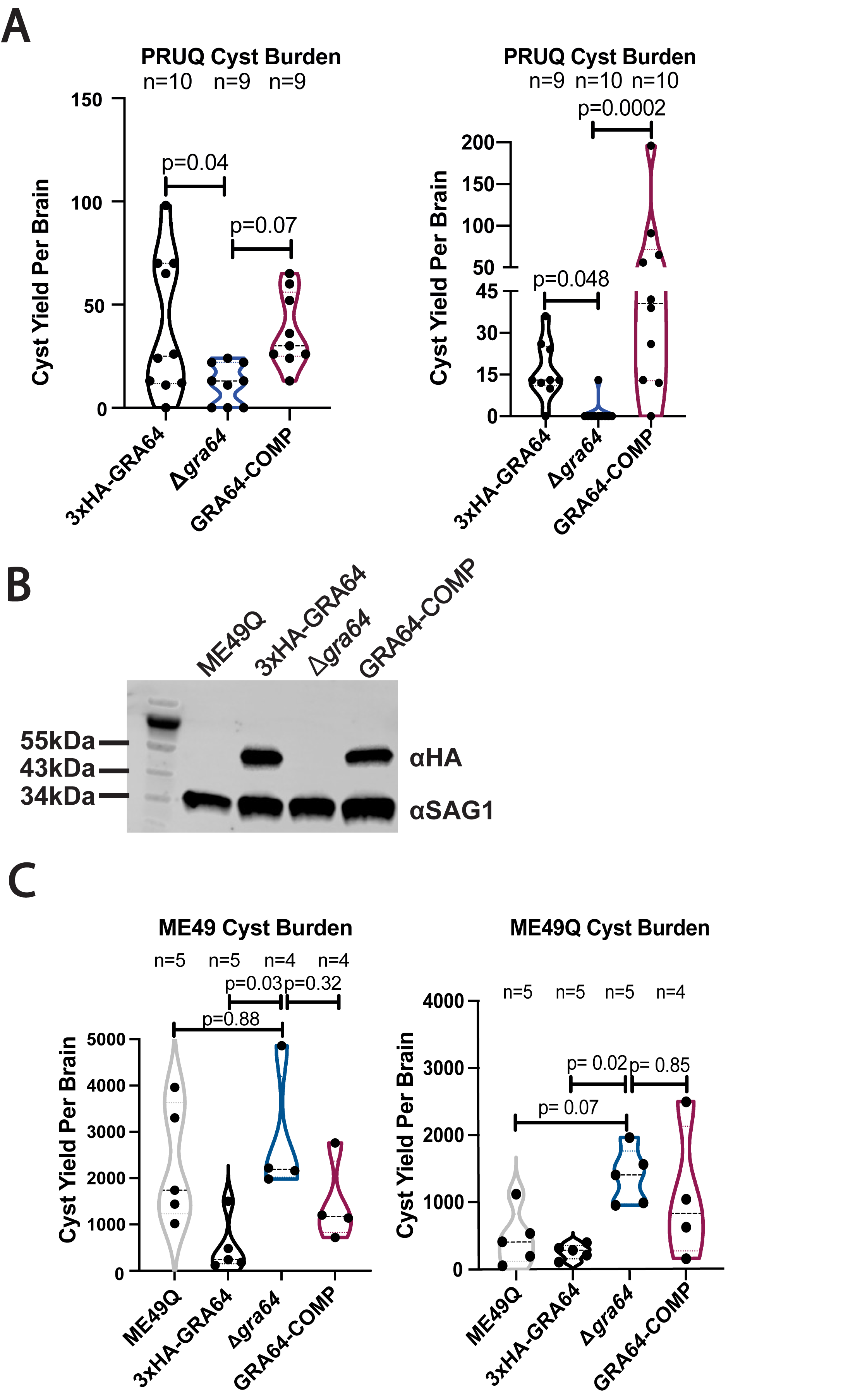

Supplement: FIG S4 [file mbio.01442-22-s0006.tif]

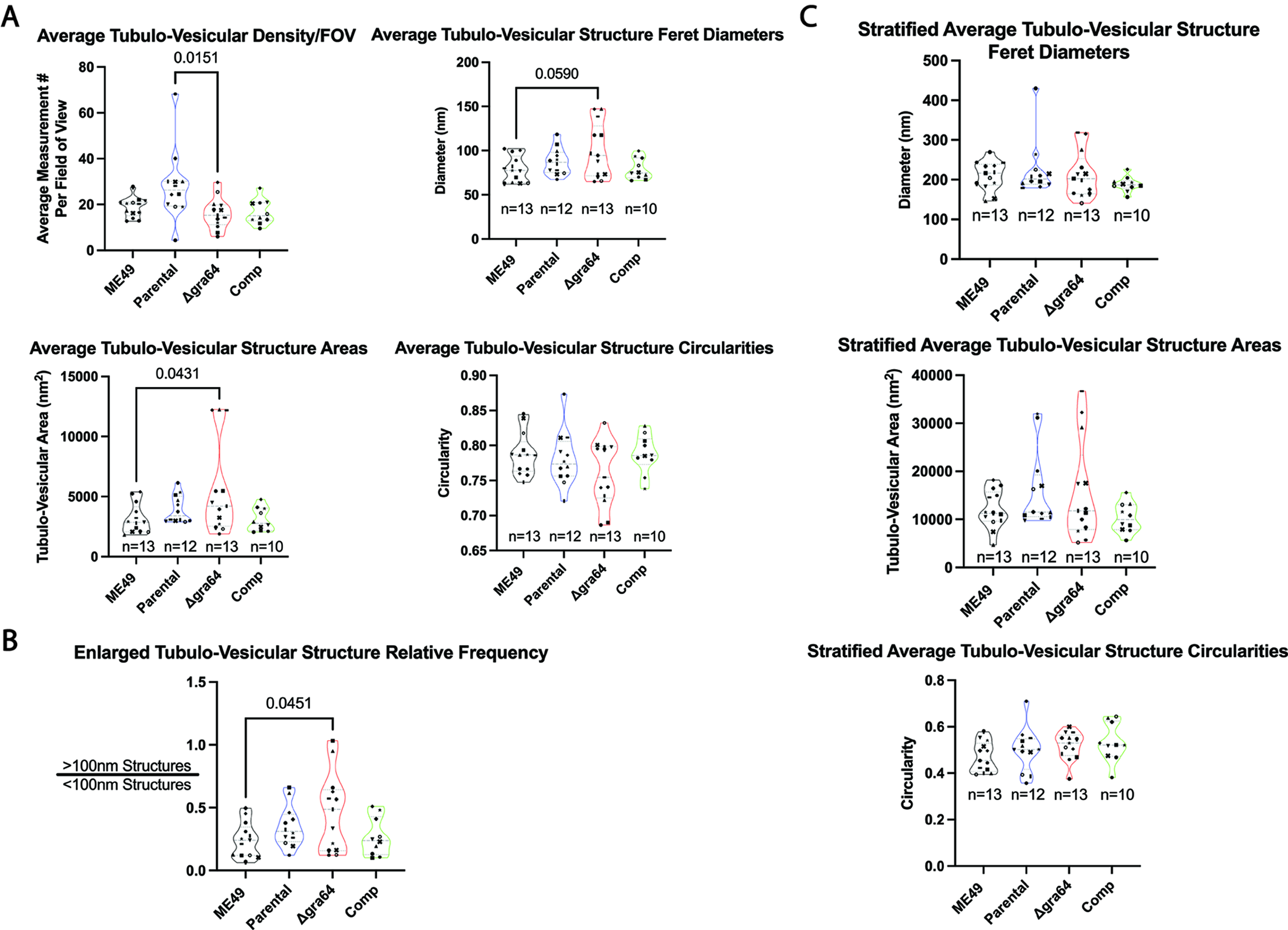

Supplement: FIG S6 [file mbio.01442-22-s0008.tif]
